# Stromal oncostatin M cytokine promotes breast cancer progression by reprogramming the tumor microenvironment

**DOI:** 10.1172/JCI148667

**Published:** 2022-04-01

**Authors:** Angela M. Araujo, Andrea Abaurrea, Peio Azcoaga, Joanna I. López-Velazco, Sara Manzano, Javier Rodriguez, Ricardo Rezola, Leire Egia-Mendikute, Fátima Valdés-Mora, Juana M. Flores, Liam Jenkins, Laura Pulido, Iñaki Osorio-Querejeta, Patricia Fernández-Nogueira, Nicola Ferrari, Cristina Viera, Natalia Martín-Martín, Alexandar Tzankov, Serenella Eppenberger-Castori, Isabel Alvarez-Lopez, Ander Urruticoechea, Paloma Bragado, Nicholas Coleman, Asís Palazón, Arkaitz Carracedo, David Gallego-Ortega, Fernando Calvo, Clare M. Isacke, María M. Caffarel, Charles H. Lawrie

**Affiliations:** 1 Biodonostia Health Research Institute, San Sebastian, Spain.; 2 Instituto de Biomedicina y Biotecnología de Cantabria, Santander, Spain.; 3 Gipuzkoa Cancer Unit, OSI Donostialdea - Onkologikoa Foundation, San Sebastian, Spain.; 4 Center for Cooperative Research in Biosciences (CIC bioGUNE), Basque Research and Technology Alliance (BRTA), Bizkaia Technology Park, Derio, Spain.; 5 Cancer Epigenetic Biology and Therapeutics Laboratory, Children’s Cancer Institute, Sydney, New South Wales, Australia.; 6 School of Women’s and Children’s Health, Faculty of Medicine, University of New South Wales, Sydney, New South Wales, Australia.; 7 Department of Animal Medicine and Surgery, Complutense University of Madrid, Madrid, Spain.; 8 The Breast Cancer Now Toby Robins Research Centre, The Institute of Cancer Research, London, United Kingdom.; 9 Department of Biochemistry and Molecular Biomedicine, Institute of Biomedicine and; 10 Department of Biomedicine, School of Medicine, University of Barcelona, Barcelona, Spain.; 11 Tumour Microenvironment Lab, The Institute of Cancer Research, London, United Kingdom.; 12 CIBERONC (Centro de Investigación Biomédica en Red de Cáncer), Madrid, Spain.; 13 Traslational Prostate Cancer Research Lab, CIC bioGUNE-Basurto, Biocruces Bizkaia Health Research Institute, Bizkaia, Spain.; 14 Institute of Medical Genetics and Pathology, University Hospital, Basel, Switzerland.; 15 Department of Biochemistry and Molecular Biology, Faculty of Pharmacy, Complutense University of Madrid, Madrid, Spain.; 16 Health Research Institute of the Hospital Clínico San Carlos, Madrid, Spain.; 17 Department of Pathology, University of Cambridge, Cambridge, United Kingdom.; 18 IKERBASQUE, Basque Foundation for Science, Bilbao, Spain.; 19 Department of Biochemistry and Molecular Biology, Faculty of Science and Technology, University of the Basque Country, Bilbao, Spain.; 20 Tumour Development Laboratory, The Kinghorn Cancer Centre, Garvan Institute of Medical Research, New South Wales, Sydney, Australia.; 21 St. Vincent’s Clinical School, Faculty of Medicine, University of New South Wales, Sydney, New South Wales, Australia.; 22 School of Biomedical Engineering, Faculty of Engineering and IT, University of Technology Sydney, Sydney, New South Wales, Australia.; 23 Radcliffe Department of Medicine, University of Oxford, Oxford, United Kingdom.

**Keywords:** Inflammation, Oncology, Breast cancer, Chemokines, Cytokines

## Abstract

The tumor microenvironment (TME) is reprogrammed by cancer cells and participates in all stages of tumor progression. The contribution of stromal cells to the reprogramming of the TME is not well understood. Here, we provide evidence of the role of the cytokine oncostatin M (OSM) as central node for multicellular interactions between immune and nonimmune stromal cells and the epithelial cancer cell compartment. OSM receptor (OSMR) deletion in a multistage breast cancer model halted tumor progression. We ascribed causality to the stromal function of the OSM axis by demonstrating reduced tumor burden of syngeneic tumors implanted in mice lacking OSMR. Single-cell and bioinformatic analysis of murine and human breast tumors revealed that OSM expression was restricted to myeloid cells, whereas OSMR was detected predominantly in fibroblasts and, to a lower extent, cancer cells. Myeloid-derived OSM reprogrammed fibroblasts to a more contractile and tumorigenic phenotype and elicited the secretion of VEGF and proinflammatory chemokines CXCL1 and CXCL16, leading to increased myeloid cell recruitment. Collectively, our data support the notion that the stromal OSM/OSMR axis reprograms the immune and nonimmune microenvironment and plays a key role in breast cancer progression.

## Graphical abstract

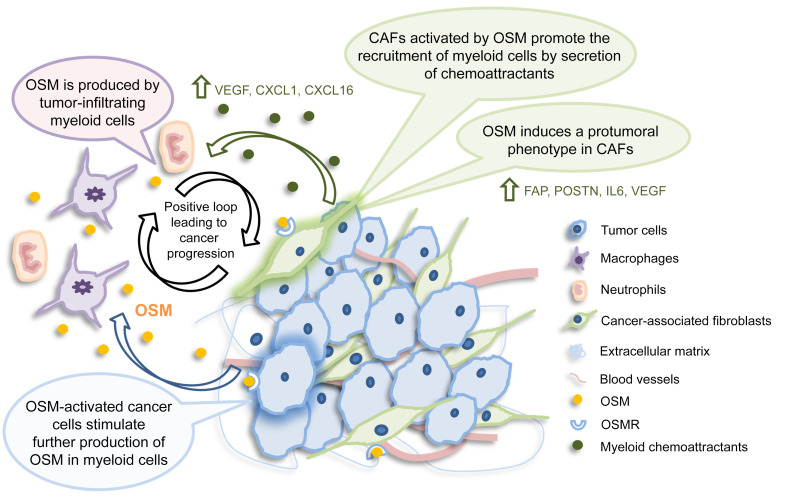

## Introduction

The tumor microenvironment (TME), composed of different cell types (e.g., fibroblasts, adipocytes, endothelial and infiltrating immune cells), harbors complex cell interactions that are often manipulated and hijacked by tumor cells in every step of cancer progression (1). Tumor cells corrupt the local microenvironment and promote the recruitment of primarily immune-suppressor cells from circulation (1). In addition, growth factors released by cancer and stromal cells (including serum growth factors) have an important role in tumor proliferation and malignant progression (2). However, the contribution of stromal cells to the reprogramming of the TME is poorly understood. Cancer-associated fibroblasts (CAFs) are a key cell population in the tumor stroma. Apart from their very well-known functions in matrix deposition and extracellular matrix remodeling, CAFs have recently been shown to interact with the immune system by responding to and secreting chemokines and cytokines (3). They differ from non-CAFs in multiple aspects and have distinctive properties, including a particular cytokine and chemokine secretory profile (4). However, CAFs are very heterogeneous and different subsets of functional fibroblasts have been proposed, some with predominant secretory functions and some with a prominent matrix remodeling phenotype (3).

Here, we discovered that the cytokine oncostatin M (OSM) acts as a central regulator of the crosstalk between immune cells, CAFs, and cancer cells, and that these immune-stromal–cancer cell interactions favor breast cancer progression and metastasis. OSM belongs to the IL-6 family, which is considered one of the most important cytokine families in the process of tumorigenesis and metastasis (5). IL-6 and OSM are acute-phase mediators of inflammation mainly produced by activated leukocytes. They can activate both epithelial and stromal cells to produce a wide array of additional inflammatory mediators (6). Nevertheless, the role of OSM in the TME remains unclear. OSM was first described as an antitumoral cytokine, owing to its antiproliferative effect in melanoma and other cancer cells (7). However, in recent years, OSM has been associated with tumor progression, as it induces epithelial-mesenchymal transition (EMT), cancer stem cell–like features, cell migration, and metastasis in animal and cellular models (8, 9). High OSM receptor (OSMR) expression in clinical samples of glioblastoma, breast cancer, and cervical cancer correlates with decreased survival in those patients (10–12). However, the information regarding the role of OSM signaling in the TME is scarce, restricted to reports describing increased mRNA expression in this compartment (13, 14) and a role for OSM in macrophage M2 polarization (15).

In this study, we used samples from human primary breast tumors, transgenic and orthotopic mouse models of breast cancer, genetically modified mice lacking OSM signaling, single-cell analysis, and in vitro cultures to demonstrate that OSM is a central node for multicellular interactions within the breast TME.

## Results

### The stromal OSM/OSMR axis promotes breast cancer progression.

**Figure 1:**
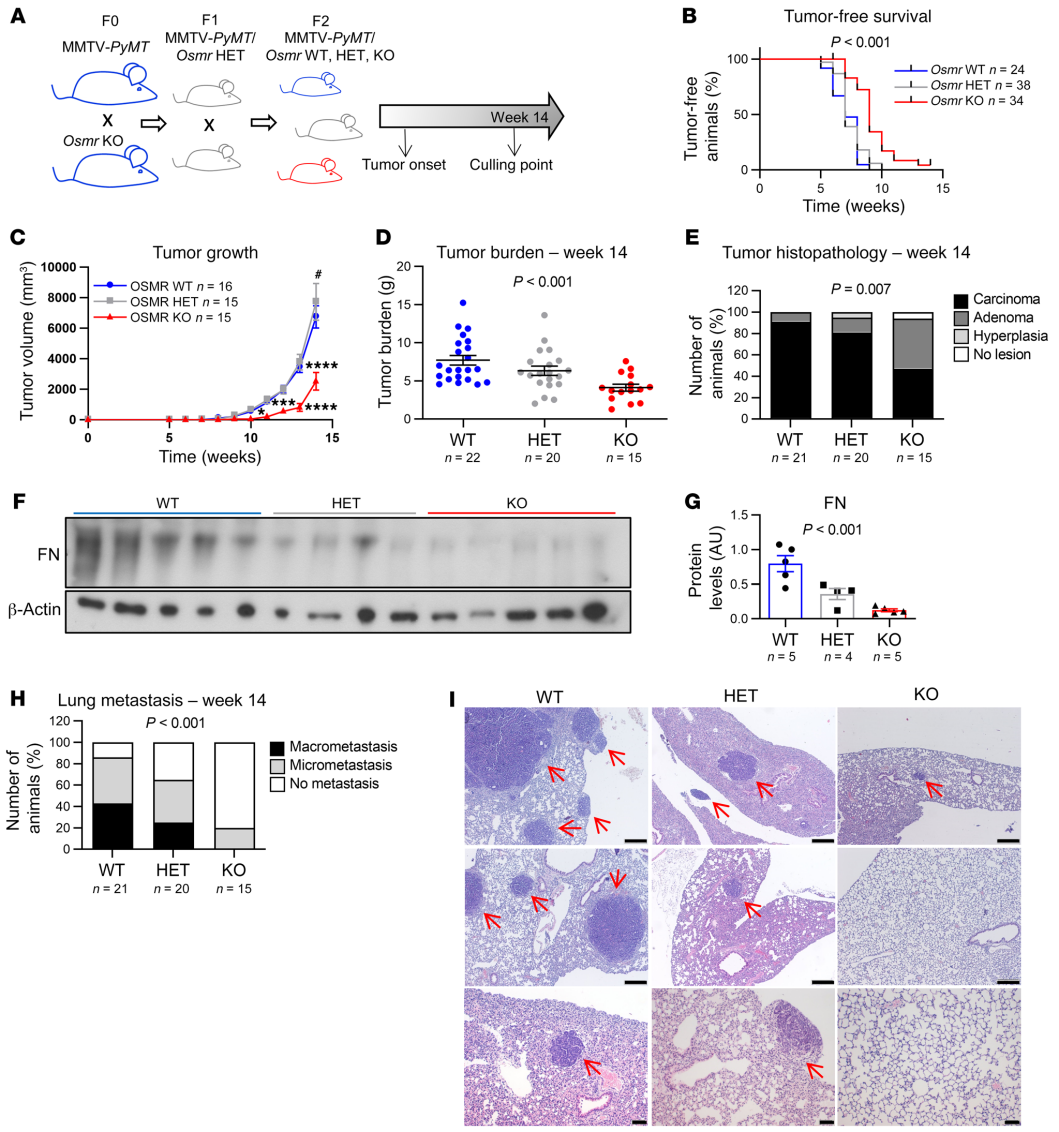
Deletion of OSMR in the MMTV-*PyMT* model hampers tumor progression and reduces metastasis. (**A**) Experimental set-up of the in vivo experiment designed to assess the importance of OSMR signaling in disease progression of the MMTV-*PyMT* mouse model. F0, F1, and F2 are different filial generations. (**B**–**D**) Kaplan-Meier curves for tumor-free survival (**B**), tumor growth (**C**), and final tumor burden (**D**) in MMTV-*PyMT* *Osmr*-WT, MMTV-*PyMT* *Osmr*-HET (heterozygous), and MMTV-*PyMT* *Osmr*-KO mice. (**E**) Histopathological analysis of tumors at week 14. Graph represents percentage of mice bearing carcinomas, adenomas, hyperplasia, and no lesions in mammary glands. *P* value was determined by comparing the number of mice with malignant carcinoma versus nonmalignant phenotypes (adenoma, hyperplasia) and no lesions using the χ^2^ test. (**F **and** G**) Western blot (**F**) and densitometric analysis (**G**) of fibronectin (FN) protein levels in tumors at week 14 from animals of the different genotypes. (**H**) Percentage of animals with lung metastases at 14 weeks of age. *P* value was determined by comparing animals with metastasis (macro and micro) versus without metastasis using the χ^2^ test. (**I**) Representative pictures of lung metastases at week 14 in MMTV-*PyMT* *Osmr*-WT, -HET, and -KO animals. Metastatic nodules are indicated with red arrows. Scale bars: 200 μm (top and middle rows) and 50 μm (bottom row). *P* values were calculated using the Mantel-Cox test (**B**), 2-way ANOVA with post hoc Dunnett’s multiple-comparison test (**C**), or 1-way ANOVA test (**D** and **G**). *P < 0.01; ***P < 0.001; ****P < 0.0001 KO vs. WT and ^#^*P* < 0.05 HET vs. WT.

First, we studied the contribution of OSM signaling in the genetic mouse model MMTV-*PyMT*, which is widely used to study breast cancer progression in a fully competent TME and immune system (16). We crossed *Osmr*-deficient mice (*Osmr*-KO) with MMTV-*PyMT* as illustrated by the experimental scheme in Figure 1A. *Osmr*-KO mice are viable but show mild defects in acute inflammation, liver regeneration, thymic hypoplasia, and net metabolism of bone and fat (17), suggesting that OSMR deficiency is not completely compensated. MMTV-*PyMT* *Osmr*-KO females showed a significant delay in tumor onset, tumor growth, and a reduced tumor burden at 14 weeks of age (Figure 1, B–D, and Supplemental Figure 1, A–D; supplemental material available online with this article; https://doi.org/10.1172/JCI148667DS1). Importantly, OSMR deletion also reduced the malignancy of the tumors, assessed by histopathological analysis, as it reduced the percentage of mice with malignant carcinomas and increased the proportion of mice with premalignant adenomas/mammary intraepithelial neoplasia (MIN) or no tumors (Figure 1E and Supplemental Figure 1E; *P* value = 0.007 for χ^2^ test comparing malignant lesions versus premalignant lesions or no lesions). Interestingly, when compared with their controls, tumors in *Osmr*-KO mice showed decreased levels of the extracellular matrix protein fibronectin, predominantly produced by CAFs (4) (Figure 1, F and G), and increased levels of apoptosis but a similar degree of proliferation (Supplemental Figure 1, F and G). Finally, OSMR deficiency produced a remarkable reduction in the percentage of animals with lung metastasis (Figure 1, H and I).

**Figure 2:**
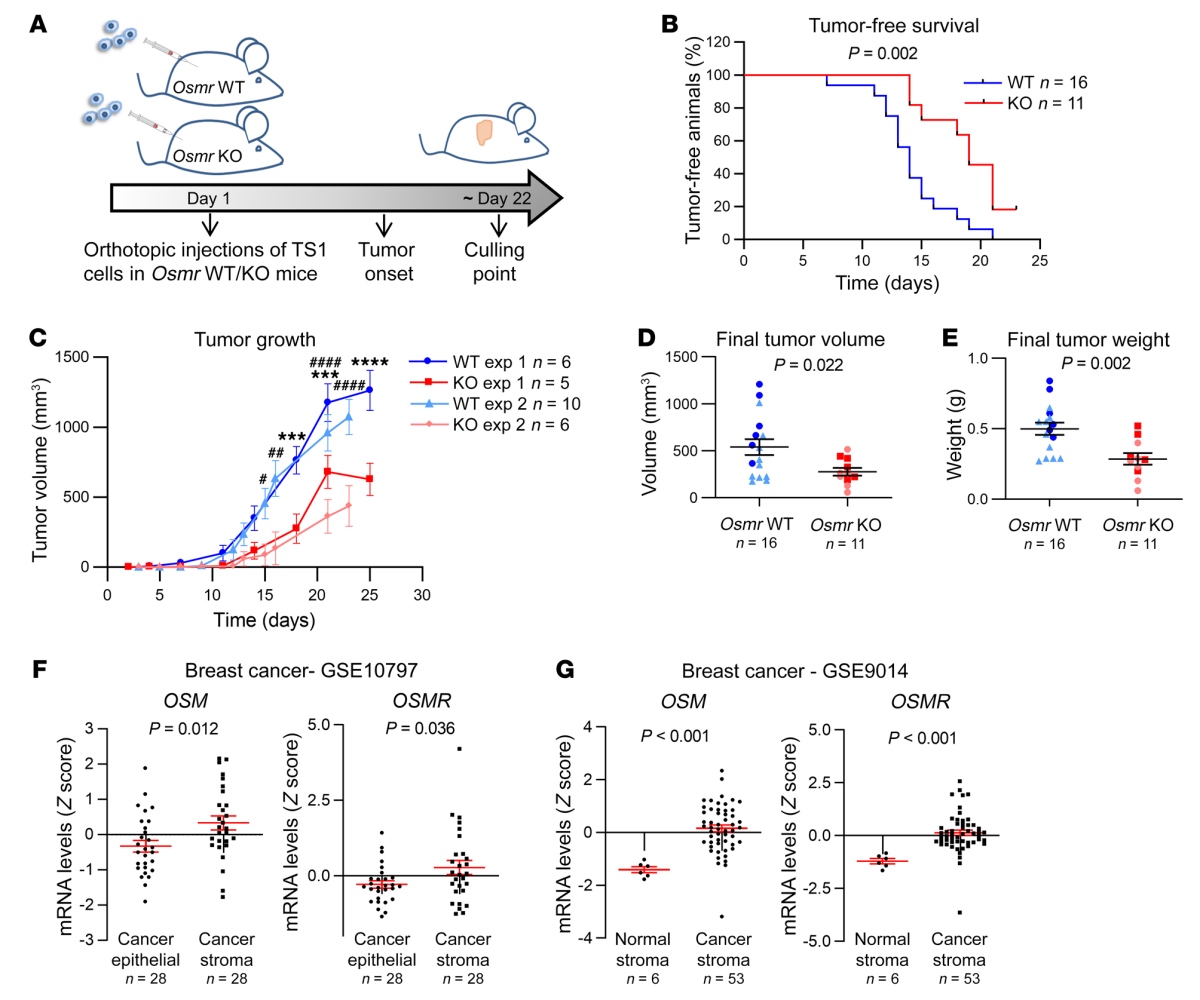
The stromal OSM/OSMR axis promotes breast cancer progression. (**A**) Experimental setup of the in vivo experiment designed to assess the importance of OSMR signaling in the tumor microenvironment, in which TS1 cells were orthotopically injected into the mammary fat pad of *Osmr*-WT and -KO mice. (**B**–**E**) Kaplan-Meier curves for tumor-free survival (**B**), tumor growth (**C**), and final tumor volume (**D**) and weight (**E**) after dissection of orthotopic tumors described in **A**. Two independent experiments were performed, and the results were combined in** B**,** D**, and** E**. (**F **and** G**) *OSM* and *OSMR* mRNA expression in paired cancer epithelial versus cancer stroma (**F**, GSE10797) and normal stroma versus cancer stroma breast cancer samples (**G**, GSE9014). Data were downloaded from NCBI GEO data sets. *P* values were calculated using the Mantel-Cox test (**B**), 2-way ANOVA with post hoc Sidak’s multiple-comparison test (**C**), or unpaired, 2-tailed Student’s *t* test (**D**–**G**). ***P < 0.001, ****P < 0.0001 for experiment 1 and ^#^*P* < 0.05, ^##^*P* < 0.01,^####^*P* < 0.001 for experiment 2.

These results show that OSM signaling is causally associated with tumor aggressiveness but, surprisingly, by using syngeneic cancer models, we found that this association requires, at least in part, the presence of the OSM/OSMR axis in the tumor stroma. We injected TS1 cells, derived from a MMTV-*PyMT* tumor (18), orthotopically into the mammary gland of syngeneic *Osmr*-KO and wild-type (WT) control mice (Figure 2A). This model allows the assessment of the contribution of stromal OSM signaling to cancer progression, as OSMR is only depleted in the TME, while TS1 cancer cells express OSMR that can be activated by host-derived OSM (Supplemental Figure 2, A–C). Depletion of OSMR in the TME resulted in delayed tumor onset and tumor growth (Figure 2, B–E, and Supplemental Figure 2, B and C), confirming that stromal OSMR signaling contributes to cancer progression. Conversely, OSMR depletion in cancer cells by the CRISPR/Cas9 technique did not show any effect on tumor onset and tumor growth in WT animals (Supplemental Figure 2, D–G).

Analyses of published gene expression profiles of breast cancer demonstrated that both *OSM* and *OSMR* are increased in human breast cancer stroma, compared with the cancer epithelial compartment and healthy stroma (Figure 2, F and G). A similar pattern of *OSM*/*OSMR* expression was observed in other cancer types, including colorectal and ovarian cancers (Supplemental Figure 3A). We also observed that increased *OSM* mRNA levels associated with decreased disease-free survival in the Metabric (19) and Wang (20) breast cancer data sets (Supplemental Figure 3B). Analysis of The Cancer Genome Atlas (TCGA) data by Kaplan-Meier Plotter (21) showed that high *OSM* levels were significantly associated with worse overall survival in other cancer types (Supplemental Figure 3C).

### The OSM/OSMR signaling module exhibits a distinct microenvironment-restricted expression.

**Figure 3:**
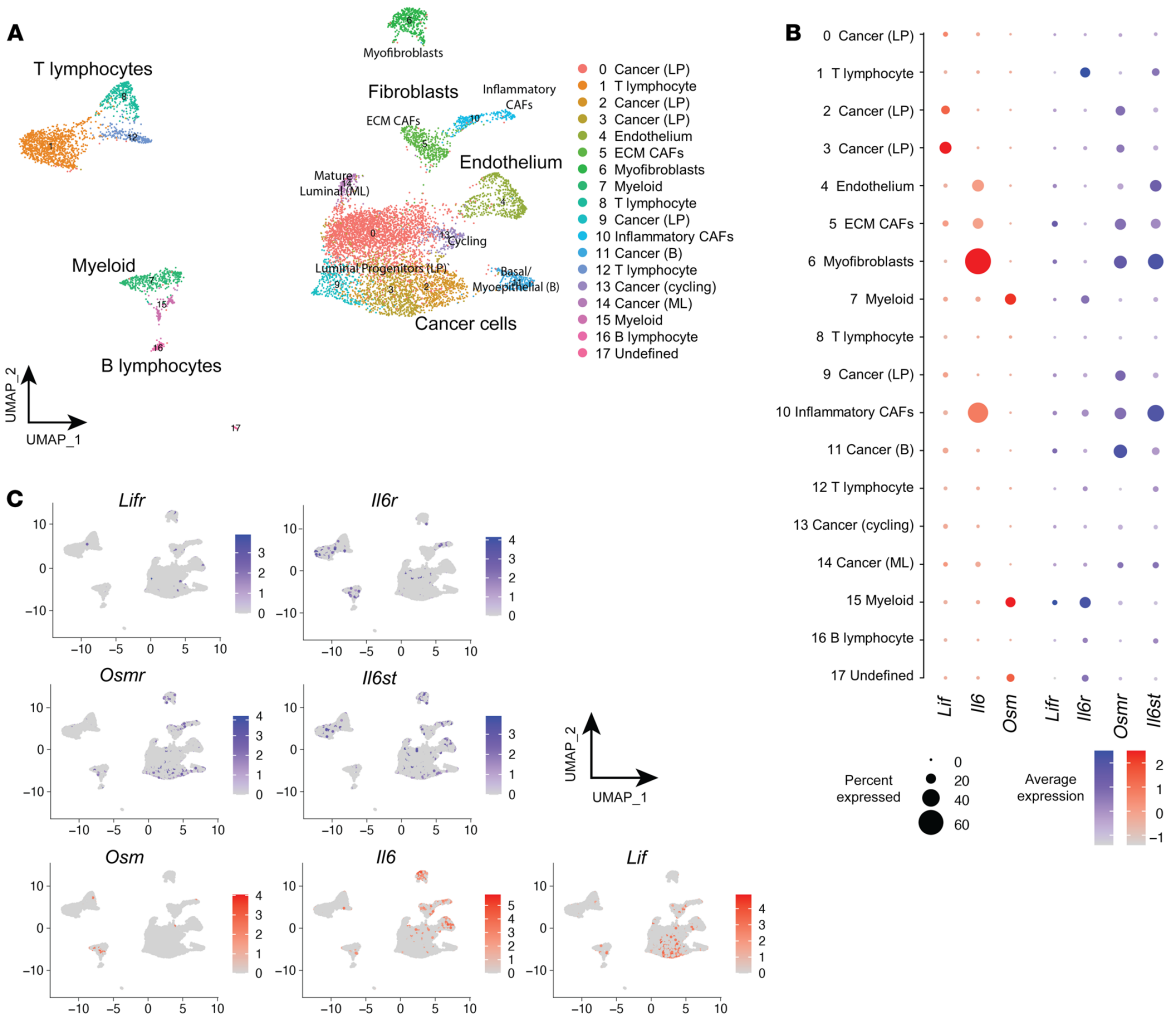
The OSM/OSMR signaling module exhibits a distinct microenvironment-restricted expression. (**A**) UMAP plot showing cell clusters defined in each of the main cell lineages. Column legend depicts the main cell lineage of origin for each cluster, showing 7 clusters of epithelial origin, 6 immune, and 4 stromal. LP, luminal progenitors; ECM, extracellular matrix; CAF, cancer-associated fibroblast; B, basal; ML, mature luminal. (**B**) Dot plot representing the expression level (red or blue jet) and the number of expressing cells (dot size) of the indicated genes in each cluster. (**C**) Feature UMAP plots showing the expression of the indicated genes in each of the main cell clusters.

**Figure 4:**
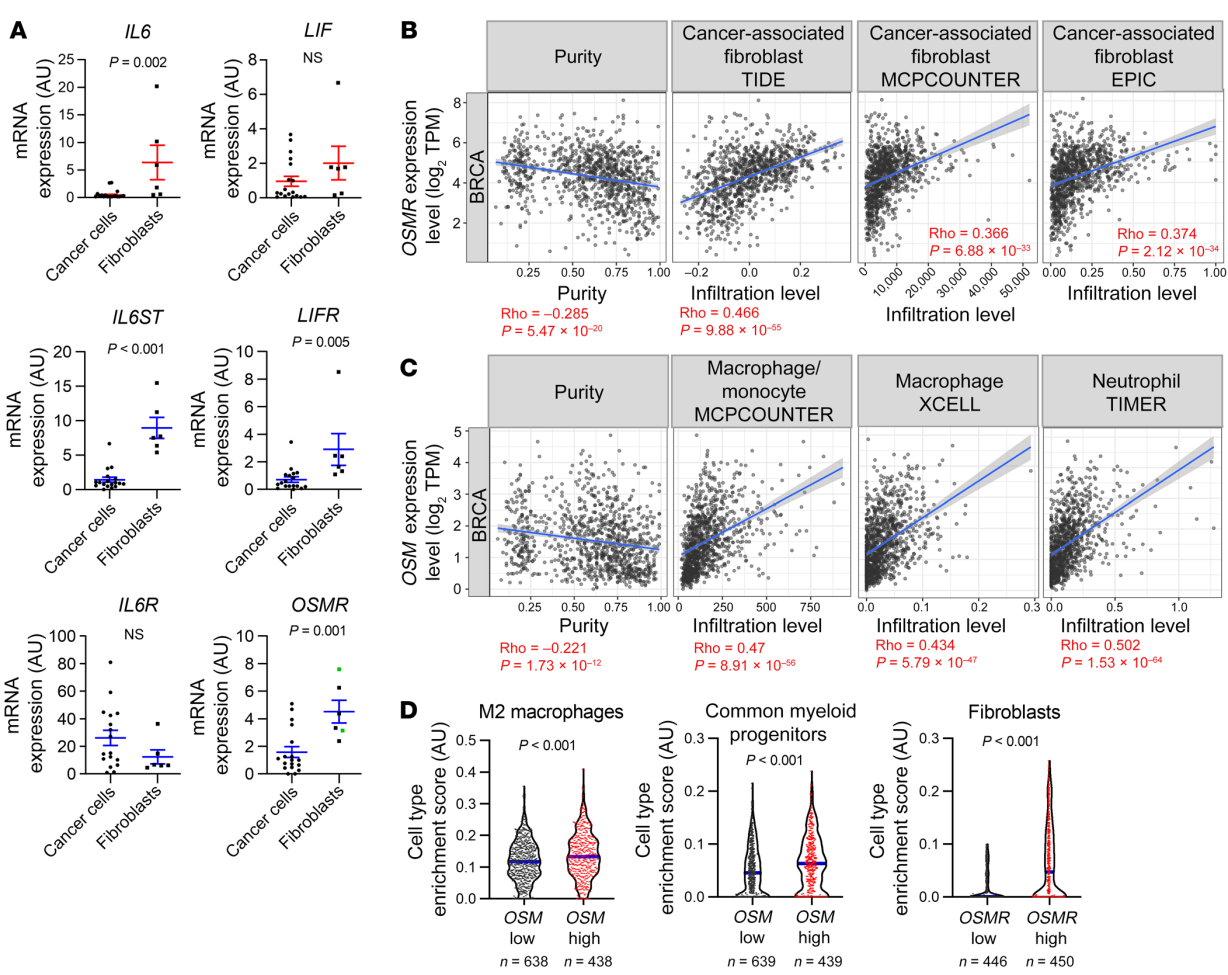
*OSM* and *OSMR* expression in human breast cancer microenvironment. (**A**) mRNA expression levels of the indicated IL-6 family members and associated receptors analyzed by RT-qPCR in a panel of breast cancer cell lines (*n =* 18) and immortalized fibroblasts (*n =* 6). In the *OSMR* graph, green and dark dots represent normal mammary fibroblasts and CAFs, respectively. *P* values were determined using the unpaired, 2-tailed Student’s *t* test. (**B **and** C**) Correlation of *OSMR* (**B**) and *OSM* (**C**) expression with tumor purity and infiltration level of indicated cell types in breast cancer samples. Data were downloaded from the TIMER web platform (*n =* 1,100). Spearman’s correlation coefficients and *P* values are shown. TPM, transcript count per million reads. (**D**) Truncated violin plots showing cell type enrichment of the indicated populations in breast tumors according to high (top quartile) or low (lowest quartile) *OSM* or *OSMR* expression. Data were obtained using the xCell web resource on 1,809 breast cancer samples from the Kaplan-Meier Plotter website. *P* values were determined using Mann-Whitney test.

As we found an unexpected contribution of the stromal OSM/OSMR axis to breast cancer progression, we performed single-cell RNA sequencing (scRNA-seq) analysis of mammary tumors from the MMTV-*PyMT* model to decipher which cells were responsible for producing OSM and expressing OSMR in the breast cancer context (Figure 3A). Our data indicate that *Osm* is almost exclusively expressed by the myeloid cell population, while *Osmr* is mainly expressed by fibroblasts and some of the cancer epithelial clusters (Figure 3, A–C). The OSM/OSMR signaling module exhibits a distinct microenvironment-restricted expression and it differs from the one observed for other cytokine-receptor pairs of the same family such as IL-6/IL-6R and LIF/LIFR (Figure 3, B and C), supporting the notion that OSM exerts distinct and unique functions from other members of the family (22). *Il6st* (*GP130*) encodes the common receptor subunit for OSM, IL-6, LIF, and other cytokines of the family and is ubiquitously expressed (Figure 3, B and C), while the expression of the other receptor subunits is more restricted and tightly regulated. Reverse transcription quantitative PCR (RT-qPCR) analysis of FACS-isolated breast TS1 orthotopic tumors (18) confirmed expression of *Osm* in the myeloid population and expression of *Osmr* in fibroblasts and in cancer cells (Supplemental Figure 4, A–C). Similar results were obtained when analyzing FACS-isolated populations of MMTV-*PyMT* tumors (Supplemental Figure 4D). An identical pattern of *OSM/OSMR* expression is maintained in the human setting, as demonstrated by RT-qPCR quantification in a large panel of human cell lines and analysis of RNA-seq data from the Human Protein Atlas (ref. 23, Figure 4A, and Supplemental Figure 5, A and B). *OSM* mRNA expression was restricted to undifferentiated and macrophage-like differentiated HL-60 cells (ref. 24 and Supplemental Figure 5A) and lymphoid and myeloid cell lines (Supplemental Figure 5B). Conversely, *OSMR* was only detected by RT-qPCR in breast cancer cells and fibroblasts, showing significantly higher expression in fibroblasts compared with epithelial cells (Figure 4A and Supplemental Figure 5A). Analysis of a battery of human cell lines (23) confirmed expression of *OSMR* only in epithelial, endothelial, and fibroblast cell lines and not in immune cell lines (Supplemental Figure 5B). To demonstrate the relevance of our previous findings in human cancer clinical data, we used the TIMER (25) and xCell (26) web resources to analyze the association between *OSM* and *OSMR* expression and TME composition in 2 different clinical breast cancer data sets (21, 27). TIMER analysis showed that *OSMR* mRNA expression significantly correlates with fibroblast enrichment in human breast cancer (Figure 4B), while *OSM* mRNA levels show the most significant associations with myeloid macrophage and neutrophil populations (Figure 4C). This analysis also showed that *OSM* and *OSMR* mRNA expression inversely correlated with tumor cell purity. The *OSMR* and *OSM* associations with fibroblast and myeloid cell infiltration, respectively, were validated by xCell in a different clinical data set (Figure 4D). A similar pattern of *OSM/OSMR* expression was observed in FACS-isolated colorectal tumors (Supplemental Figure 5C). Altogether, our data reveal that OSM and OSMR are stroma-expressed molecules, and point to paracrine OSM/OSMR signaling in cancer, as ligand and receptor are expressed by different cell types in the TME.

### OSM activates CAFs, promoting tumor progression.

**Figure 5:**
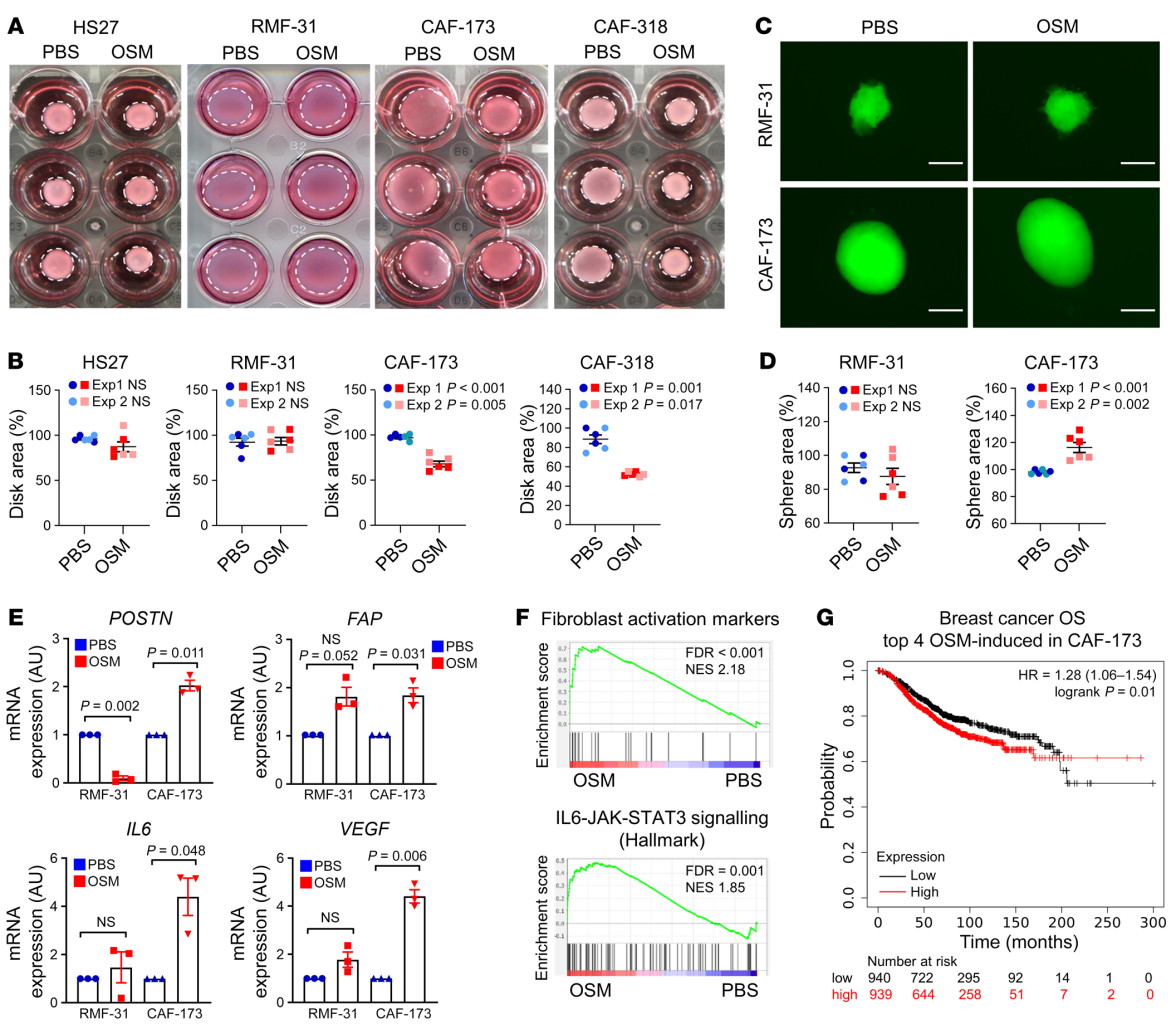
OSM activates cancer-associated fibroblasts (CAFs) in vitro, promoting their contractility and proliferation. (**A **and** B**) Representative pictures of collagen contraction assays (**A**) and quantification of collagen disk areas (**B**) of fibroblasts pretreated in monolayer with PBS or OSM. (**C **and** D**) Representative pictures (**C**) and area quantification (**D**) of 3D sphere proliferation assays of fibroblasts treated with PBS or OSM. Scale bars: 200 μm. In **B** and **D**, 2 independent experiments are plotted (experiments 1 and 2) and *P* values were calculated using the unpaired, 2-tailed Student’s *t* test. (**E**) RT-qPCR analysis of mRNA levels of activation markers in normal fibroblasts (RMF-31) and CAFs (CAF-173) cultured in 3D with PBS or OSM. *n =* 3 independent experiments. *P* values were determined using paired, 2-tailed Student’s *t* tests. (**F**) Gene set enrichment analysis (GSEA) showing enrichment of the indicated signatures in microarray data of CAF-173 treated with OSM. Data for the fibroblast activation signature were derived from Sahai et al. (4). NES, normalized enrichment score. (**G**) Kaplan-Meier curves showing overall survival (OS) for breast cancer patients according to the high or low expression in tumor samples of top 4 genes induced by OSM in CAF-173. Data were obtained using the Kaplan-Meier Plotter website. *P* value was calculated using the Mantel-Cox test and high and low expression levels were stratified by median values.

As we previously observed that fibroblasts were the cell population with higher levels of OSMR within the tumor (Figures 3 and 4 and Supplemental Figures 4 and 5), we performed complementary in vitro and in vivo experiments to assess the effect of OSMR activation in mammary CAFs and normal fibroblasts derived from human breast tumors and reduction mammoplasty surgeries, respectively (28). The ability to remodel the extracellular matrix is a hallmark of CAFs that depends on the activation of actomyosin contractility (4). Importantly, OSM treatment enhanced the capacity of CAFs (CAF-173 and CAF-318) to contract collagen matrices and, interestingly, this effect was not observed in noncancerous skin and breast fibroblasts (HS27 and RMF-31, respectively) (Figure 5, A and B). The effect of OSM in CAF contractility was blocked by pretreatment with the highly potent inhibitor of Rho-associated kinase (ROCK), Y-27632, and could not be reproduced by LIF, a member of the OSM cytokine family (Supplemental Figure 6A). In agreement, OSM activated molecular markers of actomyosin contractility (MLC2 and FAK) in CAF-173, but not in normal RMF-31 fibroblasts, and the effect was mediated by ROCK (Supplemental Figure 6, B–F). To further investigate the role of OSM in potentiating CAF activation, we selected RMF-31 to be used as a model of normal breast fibroblasts and CAF-173 as a model of CAFs. In accordance with the contractility experiments, OSM promoted the growth of 3D CAF spheroids, while it did not affect normal mammary fibroblast 3D spheroids (Figure 5, C and D). Similarly, OSM induced the expression of classical CAF markers such as *FAP*, *POSTN*, *VEGF*, and *IL6* (4), only in CAF-173 CAFs, and not in normal RMF-31 fibroblasts (Figure 5E). Of interest, *OSMR* was similarly expressed in normal fibroblasts and CAFs (Figure 4A) and the pathway was functional in both cell types, as suggested by OSM induction of *OSMR* expression in both cell lines (Supplemental Figure 7A), a classical hallmark of OSMR activation (29). Gene set enrichment analysis (GSEA) of transcriptomic data of CAF-173 treated with OSM or vehicle showed that OSM induced signatures related to fibroblast activation and JAK/STAT3 signaling, in agreement with increased STAT3 phosphorylation by OSM (Figure 5F, Supplemental Figure 7, B and C, and Supplemental Table 1). A transcriptional signature composed of the top differentially expressed genes by OSM in CAF-173 was enriched in the NCBI’s Gene Expression Omnibus breast cancer stroma data set (GEO GSE9014) compared with normal stroma (Supplemental Figure 7D). Importantly, the top 4 genes induced by OSM in CAF-173 (*SERPINB4*, *THBS1*, *RARRES1*, and *TNC*; Supplemental Figure 7E) are associated with decreased overall survival in breast cancer patients (Figure 5G). In addition, *THBS1*, *RARRES1*, and *TNC* levels correlate with *OSMR* expression in breast cancer clinical samples (Supplemental Figure 7F). These results indicate that OSM induces in CAFs the expression of promalignant genes, including fibroblast activation markers and genes associated with JAK/STAT3 signaling. Of interest, OSM induced changes in the transcriptome of CAF-173 that were different from the ones observed in OSM-activated MDA-MB-231 cancer cells (Supplemental Figure 8 and Supplemental Table 1), suggesting that OSM activates unique signaling pathways in CAFs.

**Figure 6:**
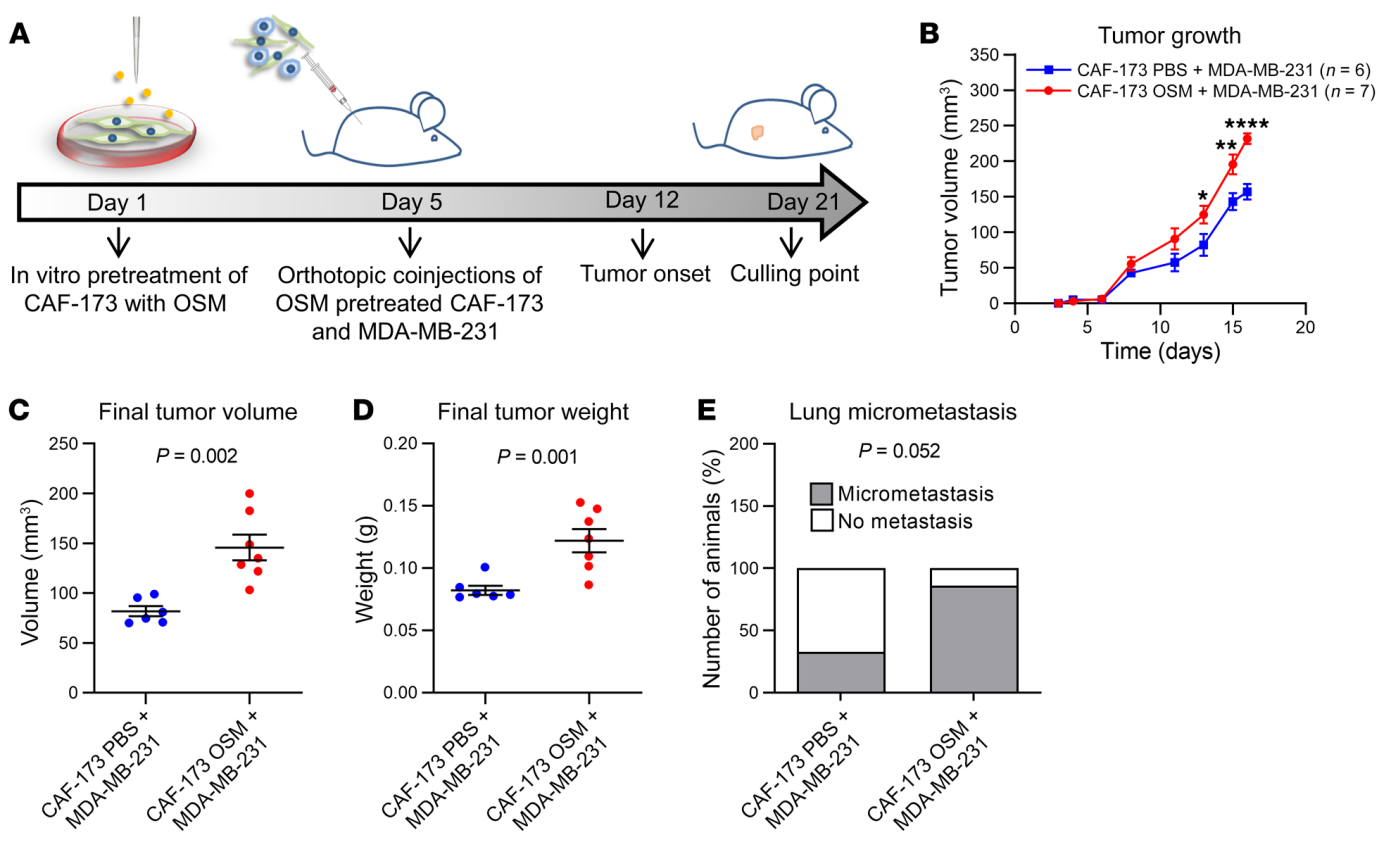
OSM activates cancer-associated fibroblasts (CAFs) in vivo, promoting tumor progression. (**A**) Experimental setup of the in vivo experiment designed to assess the contribution of OSMR activation in fibroblasts to cancer progression. CAF-173 were pretreated with OSM or PBS for 4 days prior to injection and were coinjected with MDA-MB-231 (500,000 cells per cell line) in matrigel (1:1 ratio) in the mammary gland fat pad of nude mice. *n =* 6 animals with MDA-MB-231 and PBS-treated CAF-173 cells injected, and *n =* 7 animals with MDA-MB-231 and OSM-treated CAF-173 cells injected. (**B**–**D**) Tumor growth (**B**) and final tumor volume (**C**) and weight (**D**) after dissection of orthotopic tumors described in **A**. (**E**) Percentage of animals described in **A** with lung micrometastasis assessed using qPCR analysis of genomic human *Alu* sequences. Graph represents the percentage of animals with detectable qPCR signal. *P* values were calculated using 2-way ANOVA with post hoc Sidak’s multiple-comparison test (**B**), unpaired, 2-tailed Student’s *t* test (**C** and **D**), or χ^2^ test (**E**). *P < 0.05; **P < 0.01; ****P < 0.0001.

Moreover, the changes induced by OSM in CAF-173 contributed to breast cancer malignancy, as conditioned media (CM) from OSM-treated CAF-173 stimulated cancer cell migration in vitro (Supplemental Figure 9A). To test whether the OSM-induced changes in CAFs contributed to breast cancer progression in vivo, we pretreated CAF-173 with OSM or vehicle for 4 days in vitro and orthotopically coinjected them with MDA-MB-231 breast cancer cells into athymic nude-*Foxn1*^nu^ mice, as described in the experimental timeline in Figure 6A. Activation of fibroblasts by OSM promoted tumor growth (Figure 6, B–D) and exhibited a trend to increase lung colonization (Figure 6E), as assessed by qPCR analysis of human *Alu* DNA sequences in the lungs (30). The presence of metastasis in the lung of these mice was confirmed by vimentin staining of cancer cells (Supplemental Figure 9B). Conversely, OSMR downregulation by shRNA in CAF-173 delayed tumor onset and tumor growth at early stages when coinjected with MDA-MB-231 breast cancer cells ectopically expressing human OSM (MDA-MB-231-hOSM) (Supplemental Figure 10, A–D). In addition, downregulation of OSMR in CAFs decreased *IL6* expression in tumors, suggesting that OSM is inducing the expression of similar targets in vivo (Supplemental Figure 10E). Moreover, the tumors with OSMR silencing in CAFs showed reduced levels of *GFP* (Supplemental Figure 10E), suggesting reduced levels of CAFs in this experimental group, probably due to impaired CAF proliferation upon OSMR reduction, in line with the increased size of CAF spheres observed after OSMR activation (Figure 5, C and D). Together, our data demonstrate that OSM/OSMR signaling activates CAFs and that this contributes to cancer progression.

### OSM signaling induces chemokine secretion and myeloid recruitment.

**Figure 7:**
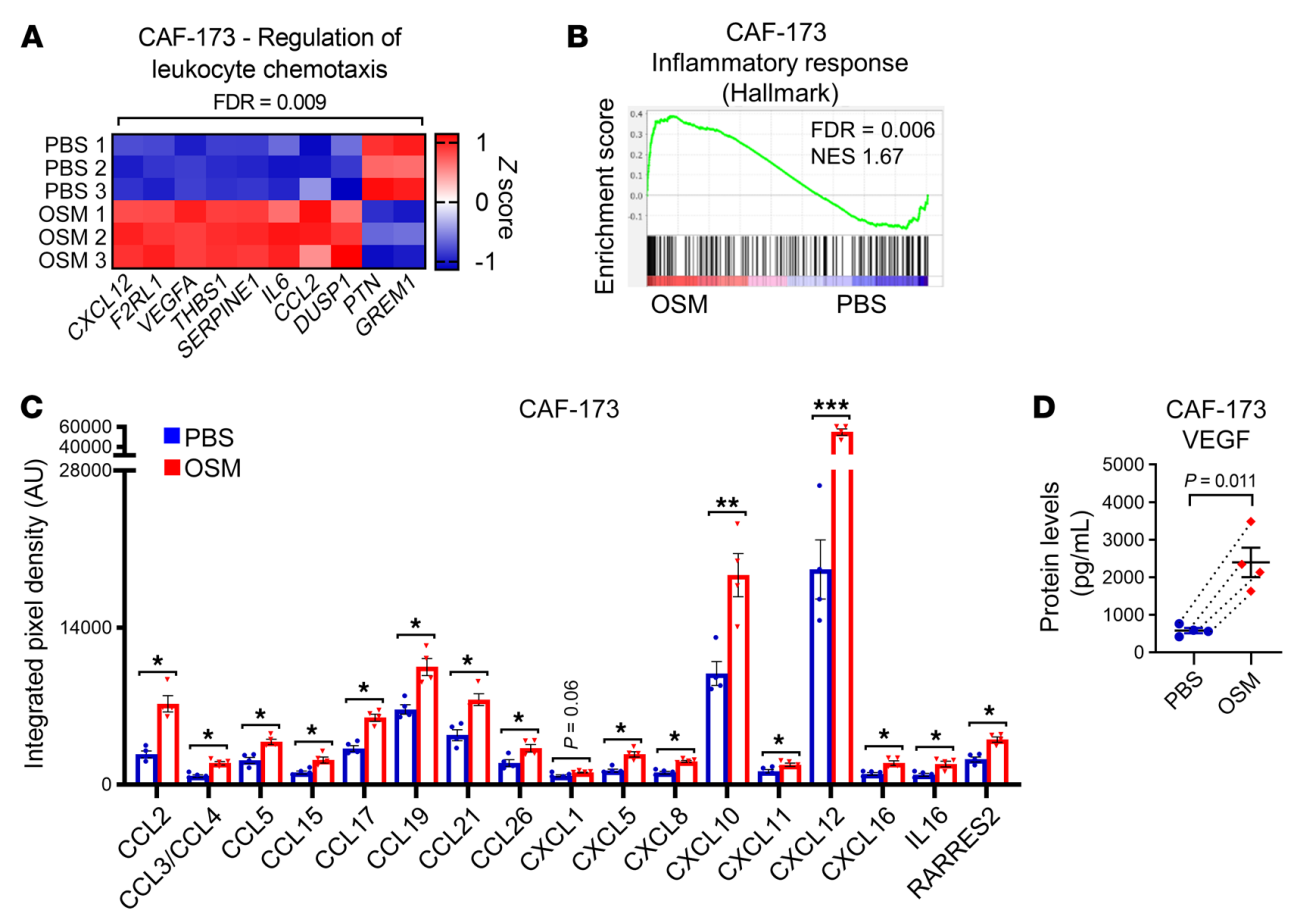
OSM/OSMR signaling in cancer-associated fibroblasts (CAFs) induces cytokine secretion. (**A**) Heatmap showing normalized mRNA expression of genes induced by OSM in CAF-173 and included in the indicated Gene Ontology (GO) pathway. (**B**) Gene set enrichment analysis (GSEA) showing enrichment of inflammatory hallmark signature in microarray expression data of CAF-173 spheres treated with 30 ng/mL OSM for 4 days. NES, normalized enrichment score. (**C **and** D**) Chemokine array analysis (**C**) and VEGF levels (**D**) in conditioned media from CAF-173 treated with PBS or 30 ng/mL OSM for 72 hours. *P < 0.05, **P < 0.01, ***P < 0.001. *P* values were determined using paired, 2-tailed Student’s *t* tests; *n =* 4 independent experiments.

**Figure 8:**
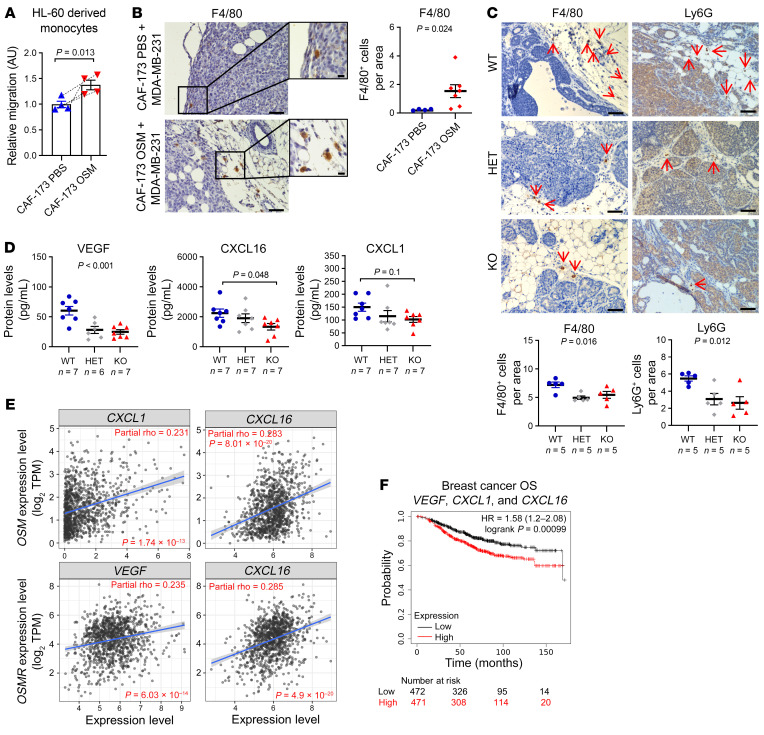
OSM/OSMR signaling induces myeloid recruitment. (**A**) Effect of conditioned media from CAF-173 treated with PBS (control) or 10 ng/mL OSM for 72 hours on HL-60–derived monocyte migration, *n =* 4 independent experiments. (**B**) Representative pictures and quantification of F4/80 immunohistochemical staining in tumors derived from MDA-MB-231/CAF-173 coinjections described in Figure 6A. Quantification was performed by manual counting of positive cells per area in a total of 12 to 19 pictures per tumor and 4 to 7 tumors per group. Scale bars: 100 μm (large pictures) and 10 μm (insets). (**C**) Representative pictures and quantification of F4/80 and Ly6G immunohistochemical staining in tumors from MMTV-*PyMT* *Osmr*-WT, -HET, and -KO mice at 14 weeks of age, described in Figure 1A. Quantification was performed by manual counting of positive cells per area in a total of 8 pictures per tumor and 5 tumors per group. Scale bars: 50 μm. (**D**) VEGF, CXCL1, and CXCL16 levels in plasma from MMTV-*PyMT* *Osmr*-WT, -HET, and -KO mice at 14 weeks of age analyzed by Luminex assay. In **A**–**D**, *P* values between the different groups were determined using paired (**A**) or unpaired (**B**) 2-tailed Student’s *t* test, 1-way ANOVA (**C**), or 1-way ANOVA with post hoc Dunnett’s multiple-comparison test (**D**). (**E**) Correlation of *OSM* and *OSMR* levels with *VEGF*, *CXCL1*, and *CXCL16* expression in breast cancer samples. Data were downloaded from the TIMER web platform (*n =* 1,100). Spearman’s correlation coefficients and *P* values are shown. TPM, transcript count per million reads. (**F**) Kaplan-Meier curves showing overall survival (OS) for breast cancer samples according to the expression of *VEGF*, *CXCL1*, and *CXCL16*. Data were downloaded from Kaplan-Meier Plotter. *P* value was determined using the Mantel-Cox test and high and low expression levels were stratified by median value.

In an attempt to understand how OSMR activation in the stroma was inducing malignancy, we probed our transcriptomic data of CAFs (CAF-173) treated with OSM. Microarray data indicated that pathways and signatures related to leukocyte chemotaxis and inflammatory response were significantly enriched by OSM (Figure 7, A and B). Interestingly, transcriptomic analysis of breast cancer cells (MDA-MB-231) activated by OSM showed enrichment of similar pathways (Supplemental Figure 11, A and B). These data suggested that, upon OSMR activation by OSM, both CAFs and cancer cells could be involved in shaping the TME by recruiting leukocytes to the tumor site. Analysis of a panel of 31 chemokines by antibody array showed that OSM induced expression of important chemoattractants (Figure 7C and Supplemental Figures 11C and 12). Some of these factors were exclusive to CAFs (mainly CXCL10 and CXCL12), others to cancer cells (mainly CXCL7 and CCL20), and some factors, such as CCL2, were common to both cell types. Vascular endothelial growth factor (VEGF) can also modulate tumor immunity by inducing macrophage and myeloid-derived suppressor cell (MDSC) recruitment (31), and we previously showed that it is an OSMR target (29). As seen in Figure 7D and Supplemental Figure 11D, VEGF levels were increased upon OSM treatment both in CAFs and tumor cells. As some of the OSM-induced chemokines are potent myeloid chemoattractants (e.g., VEGF, CCL2, and CXCL12; refs. 32, 33), we sought to determine whether OSMR activation influenced myeloid recruitment. Of interest, only CM from OSM-treated CAF-173, and not from OSM-treated MDA-MB-231 cancer cells, promoted monocyte recruitment in vitro (Figure 8A and Supplemental Figure 11E). Accordingly, activation of CAFs by OSM resulted in increased numbers of tumor-associated F4/80-positive macrophages in vivo (Figure 8B). We also investigated whether myeloid cell populations were altered in tumors after OSMR signaling abrogation and we quantified the number of F4/80-positive macrophages and Ly6G-positive myeloid cells (1, 34, 35) in MMTV-*PyMT* *Osmr*-KO and control tumors. We observed that these 2 populations were reduced in MMTV-*PyMT* *Osmr*-KO tumors compared with *Osmr*-WT tumors (Figure 8C). The decreased number of macrophages in MMTV-*PyMT* *Osmr*-KO tumors was confirmed by FACS analysis of CD45^+^CD11b^+^GR1^med^F4/80^+^ macrophages (Supplemental Figure 13, A and B). Of interest, there was no difference in the percentage of M2-like protumoral (CD206^+^) and M1-like antitumoral (CD80^+^) macrophages in those tumors (Supplemental Figure 13C), suggesting that OSMR affects macrophage recruitment without altering their polarization. We did not observe a reduction in CD45^+^CD11b^+^GR1^hi^ neutrophils in *Osmr*-KO tumors by FACS analysis (Supplemental Figure 13, A and B), suggesting that Ly6G staining in MMTV-*PyMT* *Osmr*-KO tumors may be marking neutrophils but also other myeloid-derived cells such as MDSCs (36, 37). Marker analysis showed that most of the tumor-infiltrating neutrophils in MMTV-*PyMT* *Osmr*-WT and -KO tumors exhibited a protumoral and immunosuppressive phenotype, as assessed by CXCR4 and CCR5 positivity (refs. 38, 39, and Supplemental Figure 13D). Interestingly, tumor-bearing MMTV-*PyMT* O*smr*-KO mice compared with control mice showed reduced serum VEGF and CXCL16 levels and exhibited a trend toward a decrease in CXCL1 (Figure 8D), all factors being involved in myeloid cell recruitment (31, 40, 41). Our findings are clinically relevant, as *VEGF*, *CXCL1*, and *CXCL16* mRNA expression is associated with *OSM* and/or *OSMR* levels in breast cancer patients and with decreased overall survival (Figure 8, E and F). In summary, these results show that OSM/OSMR signaling in the stroma induces cytokine secretion and myeloid cell recruitment. As OSM is mainly expressed by myeloid cells (Figures 3 and 4 and Supplemental Figures 4 and 5), our data point to the existence of a positive feedback loop where OSM signaling induces the recruitment of more myeloid cells that will in turn secrete OSM within the tumor. Intriguingly, CM from cancer cells pretreated with OSM further increased *OSM* expression in macrophage-like differentiated HL-60 cells (Supplemental Figure 13E). We did not observe this effect with CM from OSM-activated CAFs or with OSM itself. Therefore, we have discovered an unprecedented positive feed-forward loop between cancer cells, CAFs, and myeloid cells in which (a) tumor-infiltrating myeloid cells secrete OSM; (b) CAFs become activated, promoting further myeloid cell recruitment; and (c) OSM-induced secretome by cancer cells promotes sustained OSM production by myeloid cells.

**Figure 9:**
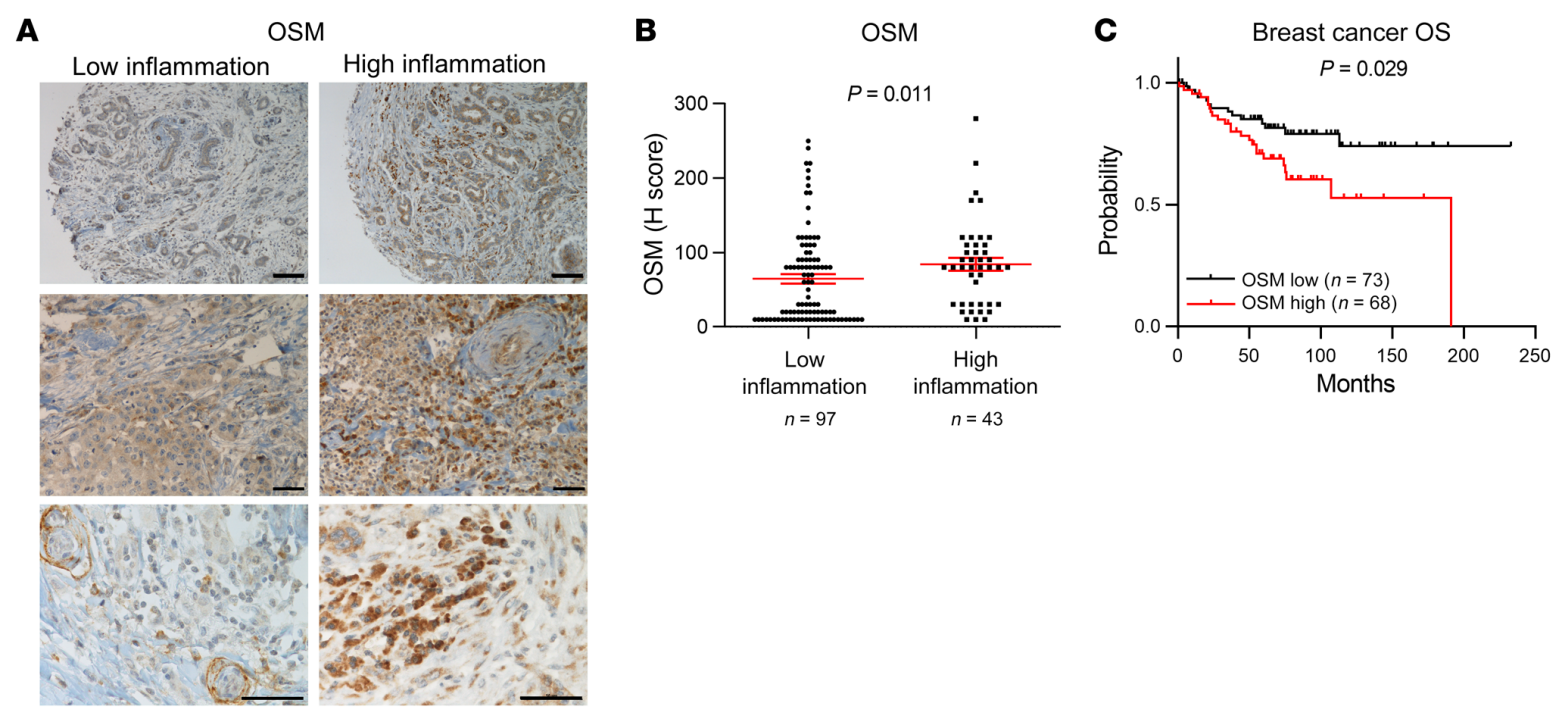
OSM expression associates with increased inflammation and decreased overall survival in human breast cancer samples. (**A **and** B**) Representative pictures (**A**) and quantification (**B**) of OSM immunohistochemical staining in samples from breast cancer patients with high and low inflammation. Scale bars: 100 μm (top row) and 50 μm (middle and bottom rows). *P* value was determined using Mann-Whitney test. (**C**) Kaplan-Meier curves showing overall survival (OS) for breast cancer patients analyzed in **A** and **B**, with high versus low OSM expression. *P* value was determined using the Mantel-Cox test and high and low expression levels were stratified by median value.

Analysis of OSM protein levels in 141 samples of early breast cancer samples confirmed the association between OSM expression and increased inflammation in a clinical setting (Figure 9, A and B). Inflammation was assessed by a pathologist as infiltration of inflammatory cells from all lymphoid and myeloid subtypes. Immunohistochemistry (IHC) confirmed that the tumor inflammatory infiltrate was composed, at least, of T cells (CD3^+^), macrophages (CD68^+^), and neutrophils (CD15^+^) (Supplemental Figure 14). We observed that OSM was mainly expressed by myeloid-like cells as determined by their larger size and more irregular shape (Figure 9A). Lymphoid cells, characterized by being smaller and round and by having a round nucleus with little cytoplasm, showed very low or negative OSM expression (Figure 9A). Importantly, high OSM protein levels were associated with decreased overall survival in this data set (*P =* 0.029; Figure 9C).

## Discussion

Cytokines are important players in inflammation, a process associated with tumor progression (42). Even cancers not directly associated with persistent infections or chronic inflammation, such as breast cancer, exhibit tumor-elicited inflammation, which has important consequences in tumor promotion, progression, and metastasis (5, 43), facilitating the acquisition of cancer hallmarks (2). Understanding how inflammatory signals orchestrate promalignant effects in the different cell compartments within the TME is key to designing new therapeutic strategies to target tumor-promoting inflammation. The oncogenic activity of inflammatory signaling factors such as IL-6 and OSM has been classically attributed to cell-intrinsic mechanisms within the cancer cell. However, our results reveal a key aspect of OSM/OSMR signaling that is instrumental for breast cancer progression beyond the epithelial compartment. Genetic and molecular analyses reveal that the tumor stroma responds to altered OSM production and signaling to influence breast cancer biology. Loss of OSMR in the nontumoral tissue hampers tumor aggressiveness, thus demonstrating that tumor cell–extrinsic OSM signaling is a pivotal factor in breast cancer progression. Our study identifies the proinflammatory cytokine OSM as a crucial mediator of the crosstalk between different cell types within the tumor by activating an intriguing protumoral “*ménage-à-trois*” between myeloid cells, CAFs, and cancer cells.

Our scRNA-seq and FACS analyses revealed that OSM and OSMR have a unique expression pattern in breast tumors, compared with other members of the IL-6 cytokine family (Figures 3 and 4 and Supplemental Figures 4 and 5). While the ligand OSM was only expressed by the myeloid cell populations, we found that the receptor OSMR was mainly expressed by fibroblasts, cancer cells, and endothelial cells. Whether there is one myeloid cell population mainly responsible for OSM production in breast cancer, or whether OSM is secreted by different immune cell types (including neutrophils, macrophages, or even circulating monocytes) remains to be determined. Of interest, a recent report identified OSM as one of the key signaling mediators of neutrophil–cancer cell interactions in prometastatic clusters of neutrophils and circulating tumor cells (44).

The cell population showing the most significant association with OSMR expression in human breast cancer samples is the CAF compartment, and our data point to an important role for this cell type in transducing OSM signaling within the TME. While the protumoral effect of OSMR activation in cancer cells has been extensively described (10, 12, 45), little is known about the effects of OSM in the tumor stroma and our results shed light on the effects of OSM signaling in CAFs. It has been previously reported that OSM and its related cytokine LIF stimulate actomyosin contractility and matrix remodeling by oral squamous cell carcinoma–derived CAFs (46, 47). However, we did not observe any effect of LIF on collagen contraction assays in our experimental setting, maybe owing to the particularities of the different protocols used. In addition to an effect on CAF contractility, we observed an increase in CAF proliferation and a proinflammatory phenotype in OSM-activated CAFs. Moreover, the secretome of OSM-activated CAFs promoted cancer cell migration. To our knowledge, this is the first report describing that OSM induces the activation of a proinflammatory transcriptional program in CAFs. In line with our results, a similar proinflammatory program was described to be activated by OSM in intestinal stromal cells in inflammatory bowel disease (48). Importantly, our results reveal that OSMR activation in CAFs promotes the recruitment of OSM-producing myeloid cells to the tumor through OSM-induced secretion of chemokines, thereby inducing a feed-forward loop. It has been shown that blocking myeloid recruitment to the premetastatic niche with anti-Ly6G antibodies inhibits metastasis (49), and impairing recruitment of tumor-associated macrophages reduces tumor incidence and metastasis (50) in MMTV-*PyMT* mice. Thus, decreased numbers of Ly6G^+^ and F4/80^+^ myeloid cells may explain, at least in part, the strong antitumoral and antimetastatic effect of OSMR depletion in the MMTV-*PyMT* cancer model. Altogether, our data show that OSMR activation in CAFs could be promoting tumor progression by different and complementary mechanisms, including increased matrix contractility and proliferation, activation of an inflammatory response, secretion of chemokines, and promotion of myeloid cell recruitment and cancer cell migration.

In summary, our results demonstrate that OSM orchestrates an intriguing protumoral crosstalk between myeloid cells, CAFs, and cancer cells that has important consequences in tumor progression. Therapies aimed at modulating inflammatory responses in the TME have been of great interest in recent years (51). Interestingly, targeting IL-6 is problematic and anti–IL-6 drugs have not yielded significant results against solid tumors in clinical trials (52, 53). Our results strongly support the notion that therapeutic targeting of OSM signaling is a valid alternative to blocking tumor-promoting inflammation in cancer that is worth exploring. The OSM/OSMR signaling module exhibits a unique microenvironment-restricted expression pattern, distinct from the rest of the members of the family, supporting the idea that targeting OSM/OSMR will potentially avoid the toxic effects of anti–IL-6 drugs. OSM-OSMR interactions could be blocked by antibody-based inhibition, a strategy that has had a major impact on cancer (54), which makes them a promising candidate for therapeutic targeting. Interestingly, humanized anti-OSM antibodies have proven to be safe and well tolerated (55) and are now in phase II clinical trials for the treatment of inflammatory diseases, such as systemic sclerosis and Crohn’s disease. Together, our findings further strengthen the case for the preclinical investigation of OSM/OSMR-blocking antibodies as a targeted anticancer therapy.

## Methods

### Mouse studies.

Generation of the congenic strain MMTV-*PyMT* *Osmr*-KO was accomplished by mating MMTV-*PyMT* mice [FVB/N-Tg(MMTV-PyVT)634Mul/J, The Jackson Laboratory], which spontaneously develop mammary tumors and lung metastases (56), with *Osmr*-KO mice (B6.129S-*Osmr*^tm1Mtan^, Riken BRC; refs. 57, 58). To transfer the transgenic *Osmr*-KO line (with a C57BL/6J background) to the genetic background of the tumor-prone animals (FVB/NJ), the *Osmr*-KO mice were previously backcrossed with FVB/NJ mice (Charles River) for 9 generations. *Osmr*-WT, -HET (heterozygous), and -KO animals used for experiments were female littermates. Tumor onset was monitored by palpation and tumors were measured once a week using a caliper, and volume was calculated as (4π/3) × (width/2)^2^ × (length/2). Animals were culled at 14 weeks of age, once tumors in the control group reached the maximum allowed size. Tumor burden was calculated by adding the volume or the weight of all the tumors from the same animal. For whole-mount analysis of early lesions, abdominal mammary glands from 9-week-old MMTV-*PyMT* *Osmr*-KO and control female mice were spread out on a glass slide, fixed overnight in Carnoy’s solution, stained with Carmine Alum, and cleared in ethanol and xylene. Pictures were taken with a Nikon D5000 at 60 mm focal length. For the generation of syngeneic orthotopic tumors, 300,000 viable murine control or *Osmr*-KO TS1 cells (derived from a MMTV-*PyMT* tumor in FVB/NJ mice; ref. 18) in growth factor–reduced (GFR) matrigel (1:1 ratio, Corning), were injected into the fourth right mammary fat pad of anesthetized (with 4% isoflurane) 6- to 8-week-old female FVB/NJ *Osmr*-KO or control mice. For the orthotopic coinjections of human MDA-MB-231 breast cancer cells and CAF-173 CAFs, cells were injected into the fourth right mammary fat pad of anesthetized (with 4% isoflurane) 6-week-old female Athymic Nude-*Foxn1*^nu^ immunocompromised mice (Envigo). In OSM activation experiments, CAF-173 were treated with 10 ng/mL OSM for 4 days, prior to coinjection with MDA-MB-231 (500,000 cells per cell line) in GFR matrigel (1:1 ratio). For OSMR knockdown experiments, 100,000 MDA-MB-231-hOSM cells and 500,000 shOSMR-infected CAF-173 were coinjected in GFR matrigel (1:1 ratio). In all mouse experiments, animals were monitored 3 times a week and tumor growth was measured using a caliper. Animals were culled once tumors reached the maximum allowed size. After animal culling, lungs were visually inspected for macroscopic metastases, and mammary glands and lungs were fixed in neutral buffered formalin solution (Sigma-Aldrich). Microscopic metastases were determined by H&E staining of formalin-fixed, paraffin-embedded sections. Tumors were divided in portions for (a) preparation of tissue sections for H&E and IHC (fixed in formalin) and (b) protein and RNA extraction (snap frozen).

### Gene expression analyses of clinical data sets and bioinformatics analyses.

Disease-free survival of patients based on *OSM* mRNA expression was calculated using data from the publicly available METABRIC (19) and Wang (20) data sets with the CANCERTOOL interface (59). Kaplan-Meier curves showing overall survival of patients from various cancer types according to the expression of different genes were obtained from the Kaplan-Meier Plotter website (21). Expression values were stratified by median. RNA-seq data from 64 cell lines was retrieved from the Human Protein Atlas (23). RNA consensus–normalized expression values were plotted for *OSM* and *OSMR* transcripts using GraphPad software. Associations between *OSMR* and *OSM* mRNA expression and infiltration of different cell types from the TME were analyzed by using xCell (26) on 1,809 breast cancer samples from Kaplan-Meier Plotter website (21) and TIMER2.0, which incorporates 1,100 breast cancer samples from TCGA (25). TIMER2.0 was also used to analyze gene expression correlations, after purity adjustment. All correlations were calculated with Spearman’s rank correlation coefficient. Gene expression analyses of human tumor stroma and epithelia were retrieved from NCBI’s GEO: Finak (GSE9014, breast; ref. 60); Casey (GSE10797, breast; ref. 61); Yeung (GSE40595, ovary; ref. 62); Nishida (GSE35602, colon; ref. 63); and Calon (GSE39396, colon; ref. 64). For Affimetrix-based arrays, probe-to-gene mapping was performed using Jetset (https://doi.org/10.1186/1471-2105-12-474), while for the rest, probes with the highest variance were selected. Unless otherwise stated, expression values for each gene were *z*-score normalized.

### scRNA-seq.

Drop-seq data set (65) raw data for MMTV-*PyMT* (WT) tumors were obtained from Valdes-Mora et al. (66). This subset was subsequently analyzed using Seurat v3.2 (67). Briefly, a total of 9,636 sequenced cells from 8 MMTV-*PyMT* tumors passed the QC filter, with less than 5% mitochondrial-to-nuclear gene content (65), and fewer than 8,000 molecules/cell, as they potentially represented cell doublets. Downstream analysis was performed according to Butler et al. (67), using 30 principal components to build a shared nearest neighbor (SNN) graph calculating *k*-nearest neighbor (Jaccard Index) for each cell, subsequent cluster calling, and UMAP dimensional reduction projection (68).

### Cell culture.

Human breast cancer–associated (CAF-173, CAF-200, CAF-220, and CAF-318) and normal (RMF-31 and RMF-39) fibroblasts were derived from human breast tumors and reduction mammoplasty surgeries, respectively, immortalized, tagged with GFP, and cultured in collagen-precoated flasks (28). The aforementioned human mammary fibroblasts, TS1 cells derived from primary tumors of the MMTV-*PyMT* mouse model (18, 56), LM2 breast cancer cells (donated by Roger Gomis, IRB, Barcelona, Spain), and HS27 skin fibroblasts (donated by Ander Izeta, IIS Biodonostia, San Sebastian, Spain) were cultured in DMEM supplemented with 10% fetal bovine serum (FBS), 1% glutamine, and 1% penicillin and streptomycin. The HL-60 promyeloblast cell line, the human embryonic kidney cell line HEK293T, and human breast cancer cell lines (MDA-MB-231, BT-549, HCC38, MDA-MB-157, SUM149PT, HCC1806, HCC70, MDA-MB-468, HCC1569, HCC1954, SK-BR-3, MDA-MB-453, CAMA-1, ZR-75-1, T47D, MCF-7, and BT-474) were purchased from American Type Culture Collection (ATCC) and cultured following ATCC instructions. All cell lines were authenticated by short-tandem-repeat profiling (Genomics Core Facility at “Alberto Sols” Biomedical Research Institute) and routinely tested for mycoplasma contamination. HL-60 differentiation into macrophages and monocytes was achieved by adding 1 nM 12-*O*-tetradecanoylphorbol-13-acetate (TPA, Sigma-Aldrich) for 24 hours and 100 nM 1,25-(OH)_2_ vitamin D3 (Sigma-Aldrich) for 5 days, respectively. Recombinant hOSM or murine OSM (R&D Systems) and human LIF (Millipore) were added to cells at 10 ng/mL unless otherwise specified.

### Generation of OSM-overexpressing and OSMR-knockdown cells.

MDA-MB-231 cells were stably transfected with 2 μg of pUNO1-hOSM expression construct (InvivoGen) using TurboFect followed by blasticidin (Sigma-Aldrich) selection at 10 μg/mL. Control transfections were performed simultaneously using 2 μg of empty vector. For generation of murine TS1 *Osmr*-KO cells, we used a previously described CRISPR/Cas9^D10A^ nickase strategy (69). OSMR-targeting guides (CTTAAAGTCTCGGGTTTCAC and GTGAAACCCGAGACTTTAAG) were cloned into an All-in-One backbone containing an EGFP-coupled Cas9^D10A^ nickase mutant (AIO-GFP, Addgene). TS1 cells were transfected by nucleofection (Amaxa 4D-Nucleofector, Lonza) with 2 μg of AIO-GFP plasmid containing OSMR-targeting guides or the empty vector, and GFP^+^ cells were subjected to single-cell FACS isolation 72 hours later. For OSMR knockdown in CAF-173 cells, pLKO-puro-shOSMR lentiviral vectors were purchased from Sigma-Aldrich (NM_003999.1-1342S21C1). Lentiviral infections were performed as previously described (70).

### Collagen gel contraction assays.

To assess the collagen remodeling capacity (71), fibroblasts were treated for 4 days with recombinant hOSM (R&D Systems) or human LIF (Millipore) at 10 ng/mL or vehicle (PBS) before being embedded (250,000 cells per matrix) in collagen matrix (2 mg/mL rat tail collagen type I, Corning, in DMEM + 10% FBS) in the presence or absence of Y-27632 (10 μM, Tocris), and seeded in triplicate or quadruplicate in 24-well plates. After polymerization, collagen gels were detached, and they were treated with OSM, LIF (both at 10 ng/mL), or vehicle. Pictures were taken 48 hours later, and the area of collagen disks was analyzed using Fiji ImageJ software.

### Cell migration assays.

MDA-MB-231 and CAF-173 cells were treated with a single dose of OSM (10 ng/mL) or vehicle (PBS) for 72 hours in serum-reduced media (2% FBS) for CM generation. For breast cancer cell migration experiments, MDA-MB-231 cells were treated with the corresponding CM (diluted 1:2) for 72 hours and subjected to migration assays for 48 hours by seeding 25,000 cells at the top of 24-well Transwell inserts (8 μm pore, Corning). FBS was used as chemoattractant. Chambers were fixed in 10% formalin (20 minutes) and stained with crystal violet solution (20 minutes). For the quantification of migrated cells, crystal violet was solubilized with 600 μL of 1% SDS (30 minutes) and absorbance was measured at 570 nm. For monocyte migration experiments, 750,000 HL-60–derived monocytes were seeded at the top of the Transwell inserts with 600 μL of the corresponding CM in the lower chamber. Cells were allowed to migrate for 3 hours and the number of migratory cells in the lower compartment was counted using a hemocytometer.

### 3D fibroblast cell cultures.

Fibroblast spheres were formed by seeding 8,000 cells/well in 96-well ultra-low attachment Corning plates (Costar). Cells were treated with 30 ng/mL OSM or PBS for 3 (for transcriptomic microarray analysis) or 4 days (for RT-qPCR, Western blot analysis, and quantification of sphere area). Pictures were taken using an EVOS FL Cell Imaging System (Thermo Fisher Scientific) and the area of the spheres was analyzed using Fiji ImageJ software. Spheres were collected for RNA and protein analyses.

### Flow cytometry.

Freshly obtained tumors and mammary glands from 14-week-old MMTV-*PyMT* *Osmr*-KO, -HET, and -WT mice were mechanically disrupted in 7 mL of digestion medium (collagenase type 1, Merck) and incubated for 1 hour at 37°C. The single-cell suspension was filtered through a 70 μm cell strainer (Falcon) and treated with ACK lysis buffer (Invitrogen) for 3 minutes at room temperature. Then, cells were stained with fluorochrome-labeled antibodies described in Supplemental Table 2 and with DAPI (1:5,000, Thermo Fisher Scientific) in FACS buffer (eBioscience). Flow cytometry analysis was performed with a BD FACSymphony flow cytometer and data were analyzed using FlowJo (BD Biosciences). The gating strategy is shown in Supplemental Figure 13B. FACS isolation of TS1 GFP^+^ cells in CRISPR/Cas9^D10A^ nickase experiments was performed with a BD FACSJazz (2B74YG) cell sorter. For FACS experiments of TS1-derived tumors, TS1 cells were injected orthotopically in FVB mice as described above, and 15 days after injection, freshly obtained TS1 tumors were dissociated into single-cell suspensions and stained with the antibodies described in Supplemental Table 2. Flow sorting was performed with a BD FACSAria II cell sorter. Gating strategy for experiments is shown in Supplemental Figure 4. A pool of 4 tumors from 4 animals was used for each sorting experiment. MMTV-*PyMT* tumors were sorted by Ferrari et al. (72). Briefly, tumor populations were separated into fibroblasts (PDGFRA^+^), cancer cells (EPCAM^+^), immune cells (CD45^+^), endothelial cells (CD31^+^), and the remaining population (negative for all markers).

### Western blotting.

Cells and tumors were lysed in RIPA buffer supplemented with protease and phosphatase inhibitors (cOmplete ULTRA Tablets, Mini, EASYpack Protease Inhibitor Cocktail; and PhosSTOP, both from Roche). Total lysates were quantified by BCA (Pierce BCA Protein Assay Kit, Thermo Fisher Scientific), resolved by SDS-PAGE, and transferred to nitrocellulose membranes. After blocking with 5% (wt/vol) nonfat dry milk in TBS-Tween, membranes were incubated with the corresponding antibodies (Supplemental Table 2) overnight at 4°C. Secondary antibodies (Supplemental Table 2) were chosen according to the species of origin of the primary antibodies and detected by an enhanced chemiluminescence system (Bio-Rad). Densitometric analysis of the relative expression of the protein of interest versus the corresponding control was performed with Fiji ImageJ software. Complete unedited blots can be found in the supplemental material.

### DNA and RNA extraction, RT-qPCR, and transcriptomic analysis.

Lung genomic DNA was extracted from frozen lungs using the QIAmp DNA mini kit (Qiagen) for qPCR analysis. RNA was obtained from snap-frozen animal tissue or cell pellets and extracted using TRIzol reagent (Invitrogen) or Recover all Total Nucleic Acid Isolation kit (Invitrogen), for RT-qPCR and microarray analysis, respectively. cDNA was obtained with the Maxima First-Strand cDNA synthesis kit (Thermo Fisher Scientific) with DNAse treatment incorporated. qPCR was performed using Power SYBR Green PCR master mix (Applied Biosystems) and oligonucleotide sequences are described in Supplemental Table 3 (all purchased from Condalab). Expression levels of genes were determined using the ΔΔCt method (73) and normalized against 3 housekeeping genes optimized for each reaction (74). Human *Alu* sequences (30) were normalized against the *18S* housekeeping gene using primers capable of recognizing both human and mouse DNA. Microarray analysis was performed using the Human Clariom S assay (Thermo Fisher Scientific). RNA quality was evaluated using the 2100 Bioanalyzer (Agilent) and microarray chips were processed on the Affymetrix GeneChip Fluidics Station 450 and Scanner 3000 7G (Affymetrix) according to standard protocols (*n =* 3 per experimental condition). Data were analyzed using the Transcriptome Analysis Console 4.0 (TAC). Genes with FDR less than 0.1 and absolute fold change greater than 2 were considered significantly modulated. GO analysis was performed using Panther (75). GSEA was performed as previously described (76). FDR less than 0.25 or 0.05 was regarded as statistically significant, depending on the type of permutations performed. We compiled the GSEA signatures used in Figures 5 and 7 and Supplemental Figures 7 and 11 from the Molecular Signatures Database (MsigDB) by the Broad Institute or they were manually curated from the literature. The gene list for each signature is publicly available at http://software.broadinstitute.org/gsea/msigdb/search.jsp, in Pein et al. (77), or in Supplemental Tables 4 and 5.

### Histopathology, IHC, and immunofluorescence analyses.

Histological analysis of murine tumors and lung metastasis was performed in H&E-stained sections. Immunohistochemical staining was performed in formalin-fixed, paraffin-embedded sections using Novolink Polymer Detection Systems (Leica). Antigen retrieval was performed using boiling 10 mM citrate buffer, pH 6.0, for 15 minutes. Endogenous peroxidase activity was inactivated by incubation with 3% hydrogen peroxide in methanol (15 minutes at room temperature). Tissue sections were incubated in a humidified chamber (overnight, 4°C) using the antibodies described in Supplemental Table 2 diluted in Tris-buffered saline (TBS). For negative controls, primary antibodies were replaced by nonimmune serum. After 3 rinses in TBS (5 minutes each), samples were incubated with the corresponding secondary antibody (Supplemental Table 2). After a 30-minute incubation, tissue sections were washed in TBS (5 minutes, 3 times) and immediately incubated for 30 minutes with streptavidin-peroxidase complex diluted 1:400 in TBS (Invitrogen). The chromogen was 3,3′-diaminobenzidine (Vector Laboratories). Nuclei were counterstained with Harris’s hematoxylin for 1 minute. Pictures were obtained using the Nikon Eclipse 80i microscope with a Nikon DS-5M camera incorporated. The number of positive cells and total cells per area was counted manually in 5 to 15 different areas of samples from 5 to 7 mice per experimental group, using Fiji ImageJ software. For immunofluorescence analysis, paraffin-embedded sections from murine lungs or cells fixed on a coverslip were permeabilized with 0.1% or 0.2% Triton X-100 and 0.01% SDS after performing antigen retrieval with citrate buffer for 15 minutes. The slides were then blocked with 3% BSA/PBS containing 3% normal goat serum and 0.1% Tween 20 for 30 minutes to 3 hours and stained overnight with the corresponding primary antibodies (Supplemental Table 2) followed by secondary antibody incubation (1 hour at room temperature). F-actin was stained with Phalloidin CruzFluor 633 Conjugate (Santa Cruz Biotechnology). Nuclei were counterstained with DAPI (Thermo Fisher Scientific). Finally, sections were mounted with Mowiol (Thermo Fisher Scientific).

### Cytokine and chemokine analysis.

Cytokine and chemokine levels were analyzed in CM from CAF-173 treated with OSM (30 ng/mL) or vehicle for 72 hours (*n =* 4), and from MDA-MB-231-hOSM and corresponding control cells (*n =* 6). A panel of 31 human chemokines was analyzed by Human Chemokine Array Kit (Proteome Profiler Array, R&D Systems), and VEGF levels were quantified by Human VEGF Quantikine ELISA Kit (R&D Systems) following the manufacturer’s instructions. Mouse VEGF, CXCL1, and CXCL16 levels in plasma from 14-week-old MMTV-*PyMT* *Osmr*-KO, -HET, and -WT mice were analyzed by mouse Premixed Multi-Analyte Kit (Magnetic Luminex Assay, R&D Systems) following the manufacturer’s instructions. Detection was carried out with the MAGPIX detector and data analysis was performed using xPOTENT software, both from R&D Systems.

### Tissue microarrays.

Formalin-fixed, paraffin-embedded blocks of 141 tumor tissues from cases surgically resected at the University Hospital Basel between 1991 and 2013, and included in tissue microarrays (TMAs), were used for analysis of OSM protein expression in human samples. Complete histopathological information (Supplemental Table 6), date and cause of death, as well as date of local and/or distant relapse were available for all the patients. TMAs were generated by punching a 1-mm spot of each sample. Tissue sections were subjected to a heat-induced antigen retrieval step prior to exposure to primary antibodies (Supplemental Table 2). Immunodetection was performed using the Roche Ventana BenchMark ULTRA IHC staining system, with DAB as the chromogen. Cases were reviewed by 2 independent pathologists and OSM staining was evaluated by the semiquantitative method of H-score (or “histo” score), used to assess the extent of immunoreactivity in tumor samples (78). Inflammation was semiquantitatively assessed by a pathologist as high or low tumor infiltration of immune cells according to their morphology.

### Data availability.

RNA-seq raw data were obtained from Valdes-Mora et al. (66) and are available in the GEO repository (GSE158677). The mRNA data sets generated during the current study are available in the GEO repository (GSE195787). Source data on uncropped Western blots are provided in the supplemental material. The gene list for the fibroblast activation signature used in Figure 5 was derived from Sahai et al. (4) and is shown in Supplemental Table 4. The gene list for the CAF-173 OSM signature used in Supplemental Figure 7 includes the 233 genes differentially upregulated in CAF-173 upon OSM treatment and can be found in Supplemental Table 5. All other data files supporting the findings of this study are available from the corresponding author upon reasonable request.

### Statistics.

Statistical analyses were performed using GraphPad Prism or SPSS software (IBM). For Gaussians distributions, the Student’s *t* test (paired or unpaired) was used to compare differences between 2 groups. Welch’s correction was applied when variances were significantly different. One- or 2-way ANOVA with post hoc Tukey’s, Dunnett’s, or Sidak’s multiple-comparison tests were used to determine differences between more than 2 independent groups. For non-Gaussian distributions, the Mann-Whitney test was performed. The χ^2^ test was used to determine differences between expected frequencies. For Kaplan-Meier analyses, the log-rank (Mantel-Cox) test was used. *P* values less than 0.05 were considered statistically significant. Unless otherwise stated, results are expressed as mean ± standard error of the mean (SEM).

### Study approval.

All patients whose samples were included in the TMAs have given written informed consent for their archival tissue to be used for scientific research in accordance with the Declaration of Helsinki, and the TMA construction was approved by the responsible local Ethical Committee EKBB (Ethikkommission beider Basel), number 361/12. All procedures involving animals were performed with the approval of the Biodonostia Animal Experimentation Committee and Gipuzkoa Regional Government, according to European official regulations.

## Author contributions

AMA, AA, PA, JR, and IOQ performed all the cellular and molecular experiments. AMA, AA, JILV, and MMC performed the animal experiments. JMF performed immunohistochemistry and analyzed mouse histopathology. JILV, SM, and LP analyzed mouse immunostaining. FVM and DGO obtained and analyzed the scRNA-seq data. AMA, LJ, LEM, and AP performed FACS isolation and FACS analyses. FC and NF provided RNA from FACS-isolated tumors. AT and SEC collected and analyzed patient data, generated the TMAs, performed immunohistochemistry, and interpreted and statistically analyzed the resulting data. RR and MMC interpreted and analyzed the TMA staining. AMA, AA, PA, NMM, FC, AC, and MMC performed bioinformatic analysis. PFN and PB generated the fibroblast cell lines. PB, CV, and AA performed and analyzed immunofluorescence staining. NC, IAL, and AU contributed to experimental design. CMI and CHL contributed to experimental design and helped with supervision of the project. AMA, AA, and MMC designed and supervised the study, analyzed the data, and wrote the manuscript. Both AMA and AA contributed equally and have the right to list their name first in their CV and should be considered co–first authors. The order of co–first authors was determined by seniority in the laboratory. All authors gave final approval to the submitted and published versions of the manuscript.

## Supplementary Material

Supplemental data

Supplemental table 1

## Version 1 02/22/2022

In-Press Preview

## Version 2 04/01/2022

Electronic publication
