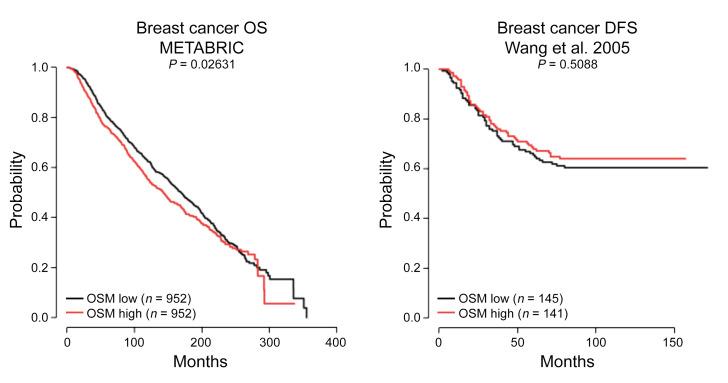# Stromal oncostatin M cytokine promotes breast cancer progression by reprogramming the tumor microenvironment

**DOI:** 10.1172/JCI165107

**Published:** 2022-10-03

**Authors:** Angela M. Araujo, Andrea Abaurrea, Peio Azcoaga, Joanna I. López-Velazco, Sara Manzano, Javier Rodriguez, Ricardo Rezola, Leire Egia-Mendikute, Fátima Valdés-Mora, Juana M. Flores, Liam Jenkins, Laura Pulido, Iñaki Osorio-Querejeta, Patricia Fernández-Nogueira, Nicola Ferrari, Cristina Viera, Natalia Martín-Martín, Alexandar Tzankov, Serenella Eppenberger-Castori, Isabel Alvarez-Lopez, Ander Urruticoechea, Paloma Bragado, Nicholas Coleman, Asís Palazón, Arkaitz Carracedo, David Gallego-Ortega, Fernando Calvo, Clare M. Isacke, María M. Caffarel, Charles H. Lawrie

Original citation: *J Clin Invest*. 2022;132(7):e148667. https://doi.org/10.1172/JCI148667

Citation for this corrigendum: *J Clin Invest*. 2022;132(19):e165107. https://doi.org/10.1172/JCI165107

The authors recently became aware that, following the recent update of Cancertool (http://genomics.cicbiogune.es/CANCERTOOL/index.html), the two panels included in Supplemental Figure 3B needed to be modified. These panels included data downloaded from Cancertool reporting the prognostic potential of OSM in two independent breast cancer patient datasets, namely METABRIC (PMID 22522925) and Wang et al. (PMID 15721472). Based on the results from the updated Cancertool data, we cannot conclude that OSM is shown to exhibit prognostic potential in the Wang dataset. In addition, the analysis of the METABRIC dataset presented in Supplemental Figure 3B pertains to overall survival, but data were inadvertently reported as showing disease-free survival. The corrected panels are shown below. Corrected sentences referring to Supplemental Figure 3B data in Results and Methods are also shown below. The online supplemental file has been updated.

## Results

We also observed that increased *OSM* mRNA levels associated with decreased overall survival in the METABRIC (19) breast cancer dataset (Supplemental Figure 3B). Of note, the prognostic analysis of *OSM* in the Wang (20) dataset did not reveal a significant association with disease-free survival (Supplemental Figure 3B).

## Methods

*Gene expression analyses of clinical datasets and bioinformatics analyses*. Overall survival (OS) and disease-free survival (DFS) data for breast cancer patients based on *OSM* mRNA expression in the METABRIC (19) and Wang (20) datasets were analyzed and represented using the CANCERTOOL interface (59) in combination with cBioPortal (www.cbioportal.org).

The authors regret the errors.

## Figures and Tables

**Figure:**