# Supplementary material for: Stromal oncostatin M cytokine promotes breast cancer progression by reprogramming the tumor microenvironment
Source: J Clin Invest. 2022 Apr 1;132(7):e148667. doi: 10.1172/JCI148667 (PMC8970678; doi:10.1172/JCI148667)

Supplemental Figure 1

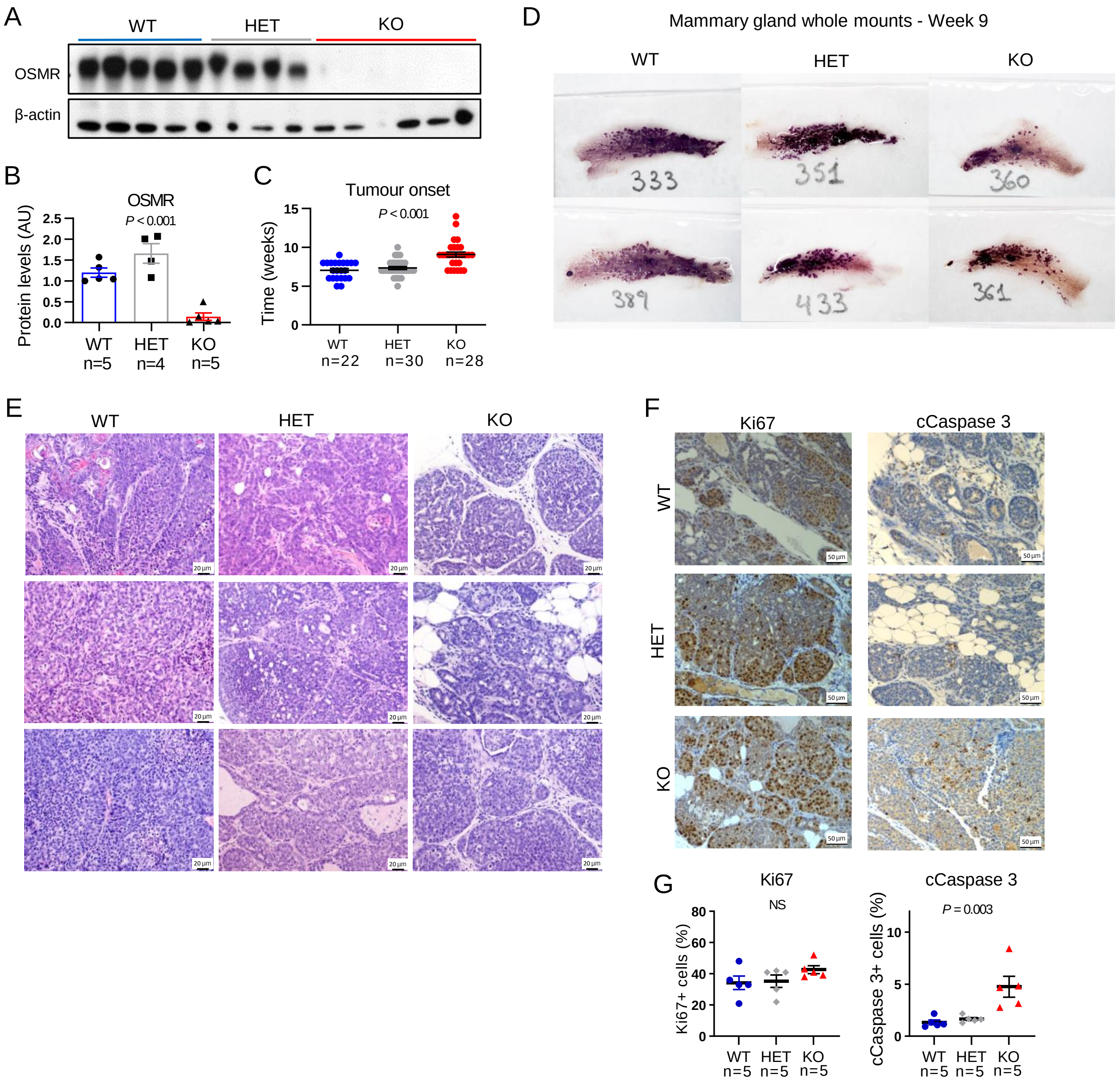

Supplemental Figure 1. Effects of OSM:OSMR signalling in cancer progression in the MMTV-*PyMT* preclinical model of breast cancer. (**A** and **B**) Western blot (**A**) and densitometric analysis (**B**) of OSMR protein levels in tumours at culling point from the different experimental groups of Figure 1A: MMTV-*PyMT:Osmr* wild-type (WT), MMTV-*PyMT:Osmr* heterozygous (HET), and MMTV-*PyMT:Osmr* knockout (KO) mice. (**C**-**E**) Tumour onset (**C**), representative pictures of whole mount staining of mammary glands at week 9 (**D**) and histopathological analysis of tumours at culling point (**E**) in the different experimental groups of Figure 1A. n= 12 WT, 6 HET, 5 KO in (**D**) and 21 WT, 20 HET, 15 KO in (**E**). (**F** and **G**) Representative pictures (**F**) and quantification (**G**) of Ki67 and active cleaved caspase 3 (cCaspase3) IHC staining in tumours at culling point of the different experimental groups of Figure 1A. Quantification was performed by manual counting of the percentage of positive tumour cells in a total of 8 pictures per tumour and 5 tumours per group. Scale bar is 20 mm (**E**) and 50 mm (**F**). *P* values were calculated using one-way ANOVA test (**B**, **C** and **G**).

Supplemental Figure 2

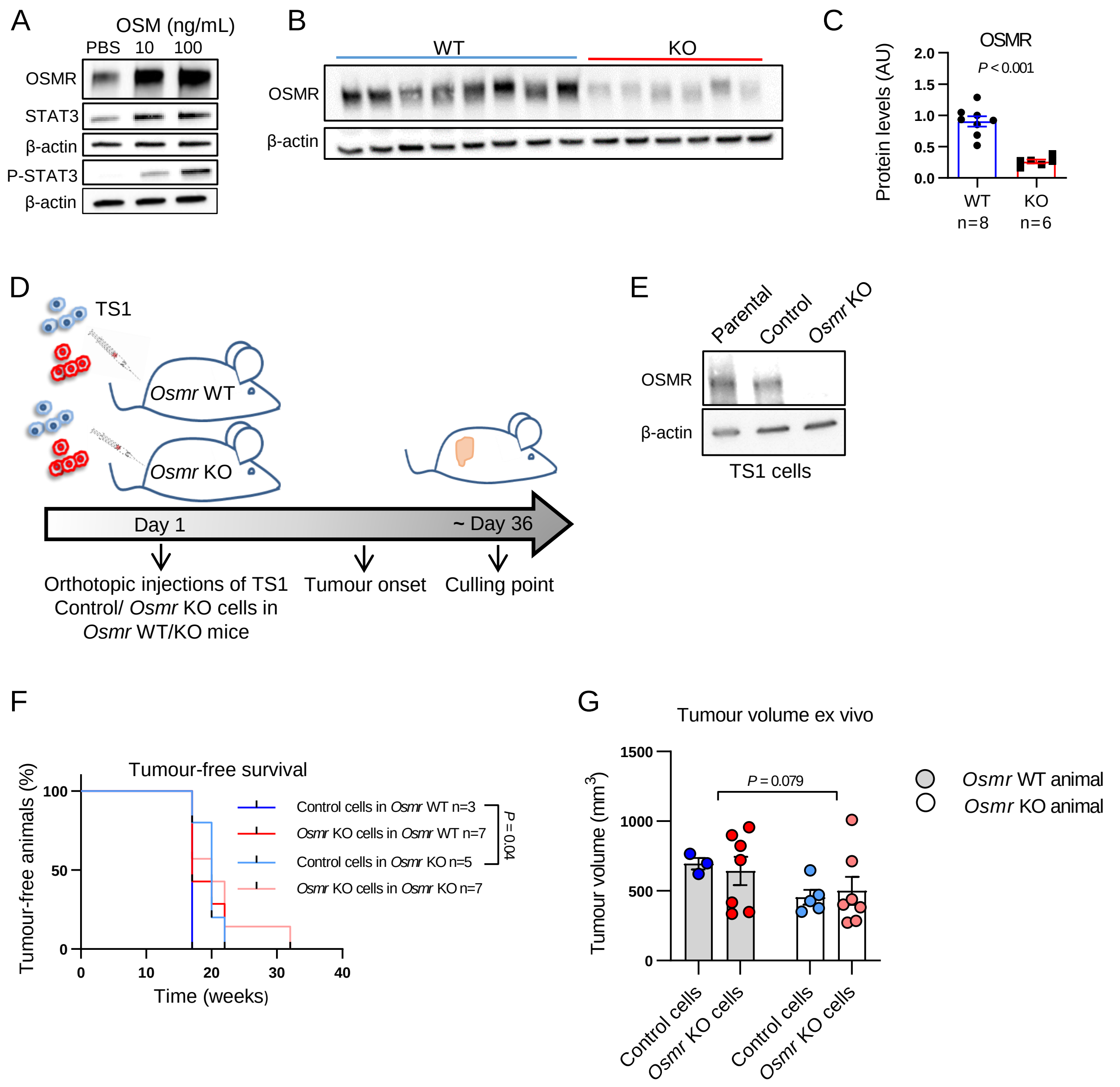

Supplemental Figure 2. Effects of stromal OSMR deletion in syngeneic models of breast cancer. (**A**) Western blot of OSMR, STAT3 and P-STAT3 protein levels in TS1 cells treated with 10 and 100 ng/mL of recombinant murine OSM for 24h. (**B** and **C**) Western blot (**B**) and densitometric analysis (**C**) of OSMR protein levels in TS1 derived-tumours from animals *Osmr* wild-type (WT, n=8) or knockout (KO, n=6) injected orthotopically with TS1 cells (experiment 2, Figure 2, A-E). OSMR expression in tumours from *Osmr* KO animals derives from the TS1 cancer cell compartment. (**D**) Experimental set-up of the in vivo experiment designed to assess the importance of OSMR signalling in cancer cells and in the tumour microenvironment, in which control and *Osmr* KO TS1 cells were orthotopically injected into the mammary fat pad of *Osmr* WT and KO mice. n=3 Osmr WT mice injected with control cells, n=7 Osmr WT mice injected with Osmr KO cells, n=5 Osmr KO mice injected with control cells, and n=7 Osmr KO mice injected with Osmr KO cells. (**E**) Western Blot of OSMR protein levels in TS1 parental, control and *Osmr* KO cells. (**F** and **G**) Kaplan-Meier curves for tumour-free survival (**F**) and final tumour volume (**G**) after dissection of orthotopic tumours described in (**D**). In **A** and **E**, one representative experiment of 2 performed is shown. *P* values were calculated using unpaired two-tailed t test (**C**), the Mantel-Cox test (**F**) or one-way ANOVA test (**G**).

Supplemental Figure 3

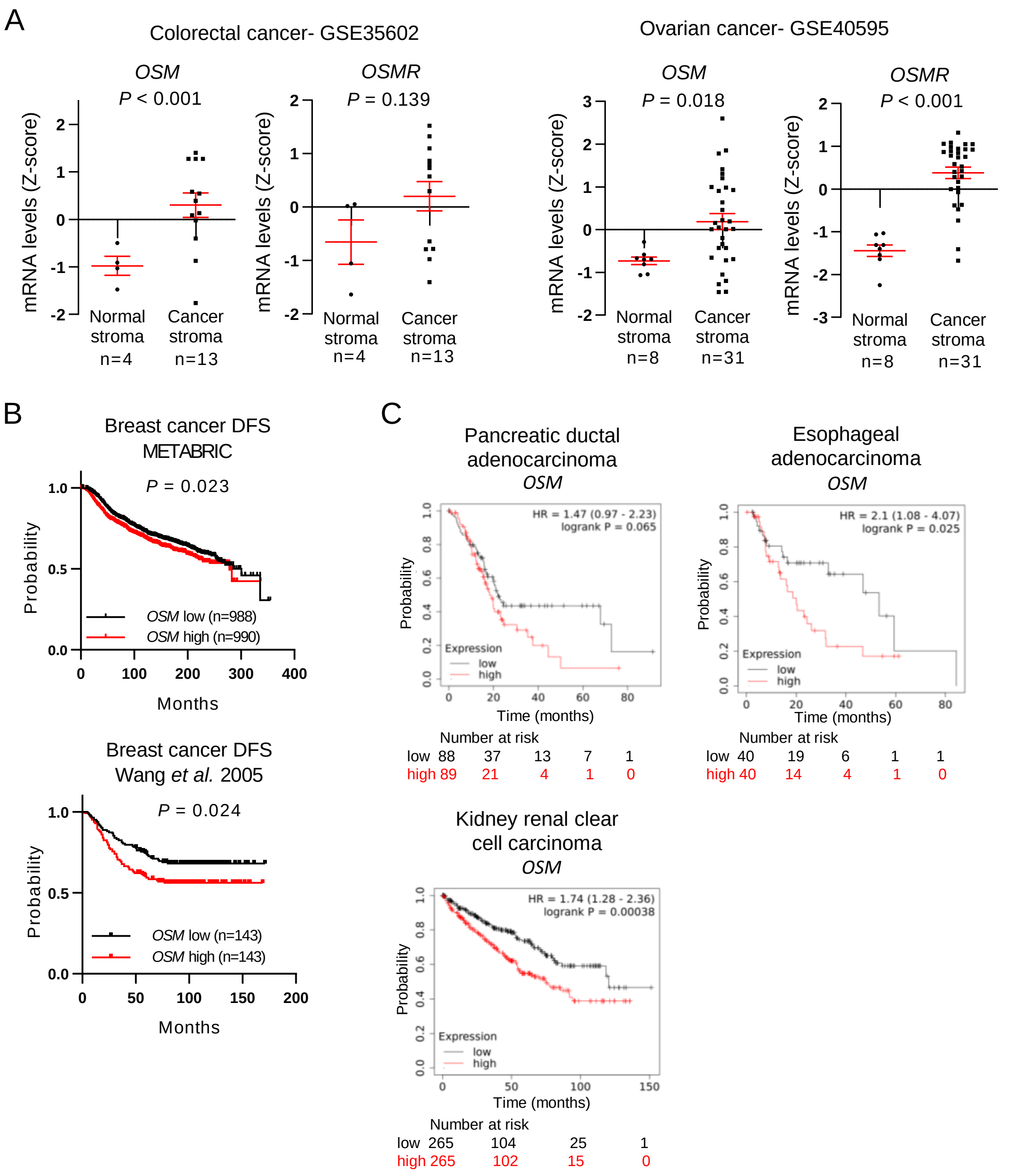

Supplemental Figure 3. Expression of OSM and OSMR in clinical samples from multiple cancer types. (**A**) *OSM* and *OSMR* mRNA expression in normal stroma versus cancer stroma samples of colorectal and ovarian cancer. Data were downloaded from GEO DataSets (GSE35602 and GSE40595). *P* values were calculated using unpaired two-tailed t test. (**B**) Kaplan-Meier curves showing disease-free survival (DFS) for breast cancer patients with high and low *OSM* expression, included in the METABRIC and Wang datasets. (**C**) Kaplan-Meier curves showing overall survival for cancer patients of the indicated tumour type with high and low *OSM* expression. Data were obtained using KM plotter website. In **B** and **C**, *P* values were determined using the Mantel-Cox test, and high and low *OSM* levels were stratified by median values.

Supplemental Figure 4

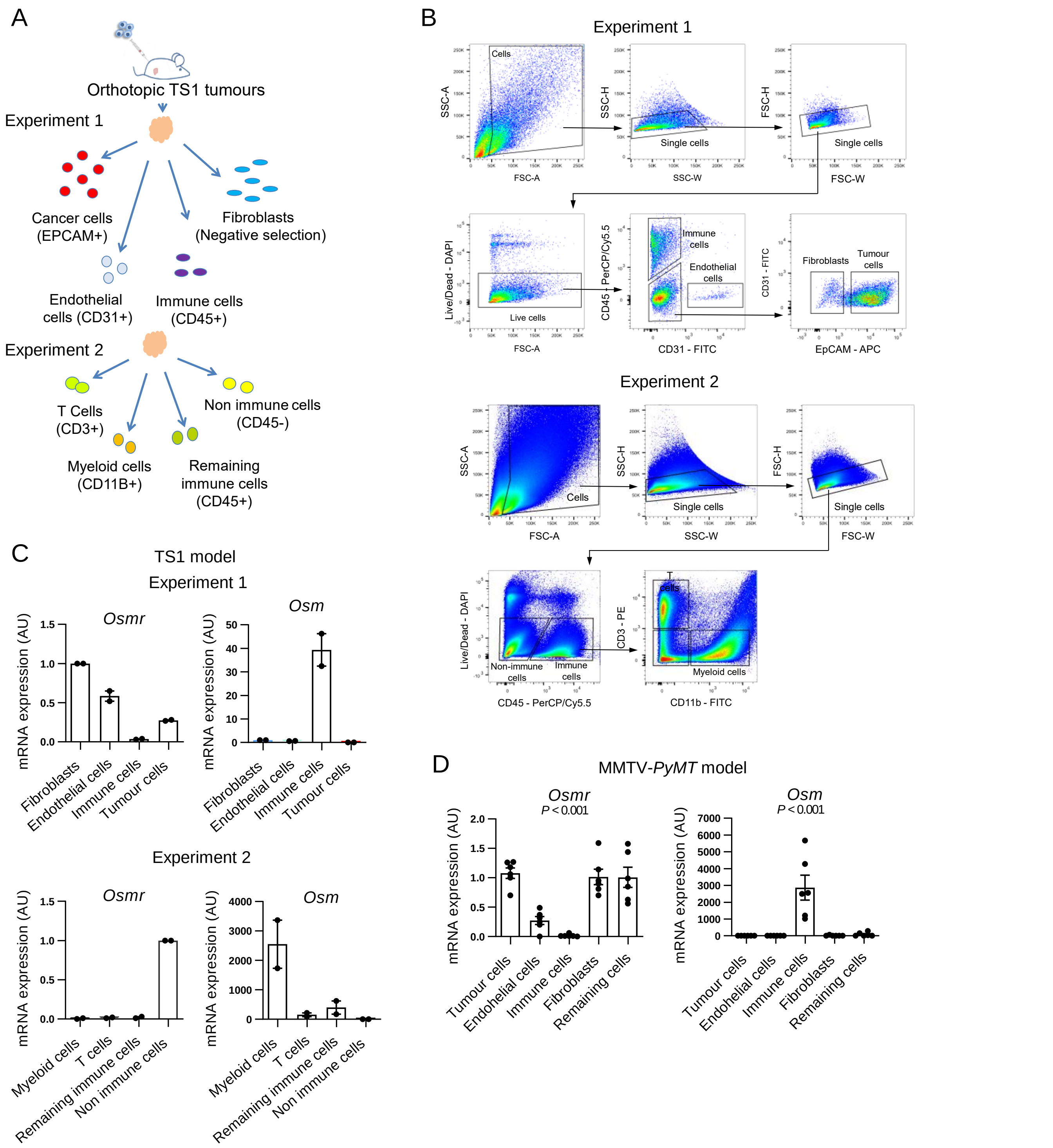

Supplemental Figure 4. *Osm* and *Osmr* expression in murine tumours. (**A** and **B**) Experimental set-up (**A**) and gating strategy (**B**) for FACS sorting experiments of TS1 orthotopic tumours. (**C** and **D**) *Osm* and *Osmr* mRNA expression levels analyzed by RT-qPCR of FACS sorted populations of TS1 orthotopic tumours (**C**) or MMTV-*PyMT* FACS sorted tumours described in Ferrari et al. (2019) (**D**). In **D**, *P* values were determined using one-way ANOVA test. In **A** and **C**, the two different experiments were performed independently, each one in a pool of 4 tumours from individual animals. Graphs represent mean of 2 technical replicates (**C**) or of 6 different tumours (**D**).

Supplemental Figure 5

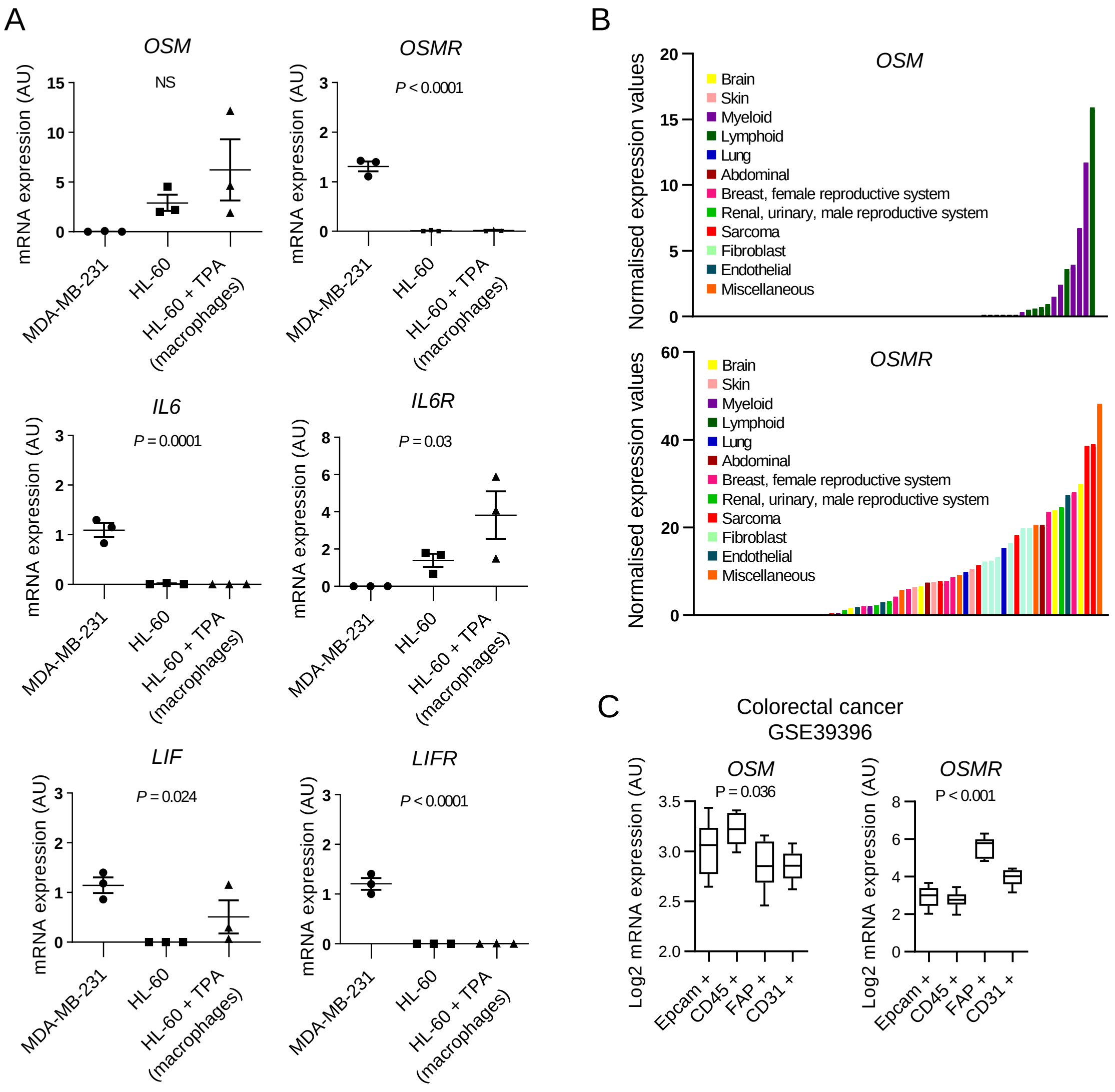

Supplemental Figure 5. *OSM* and *OSMR* expression in human cell lines and tumours. (**A**) mRNA expression levels of the indicated IL6 family members and associated receptors analyzed by RT-qPCR in MDA-MB-231 breast cancer cells, and undifferentiated and TPA-differentiated HL-60 cells. n=3 independent experiments. (**B**) *OSM* and *OSMR* mRNA relative values in a panel of 69 human cell lines from multiple anatomical sites. Data were downloaded from Human Protein Atlas. (**C**) *OSM* and *OSMR* mRNA expression in epithelial cell (Epcam^{+}), immune cell (CD45^{+}), fibroblast (FAP^{+}) and endothelial cell (CD31^{+}) FACS-sorted populations from colorectal cancer samples (n=6). Boxplot with Tukey whiskers is shown, bounds of the boxes represent the 25th and 75th percentiles, and lines within the boxes indicate the median. Data were downloaded from GSE39396 GEO DataSets. *P* values were determined using one-way ANOVA test.

Supplemental Figure 6

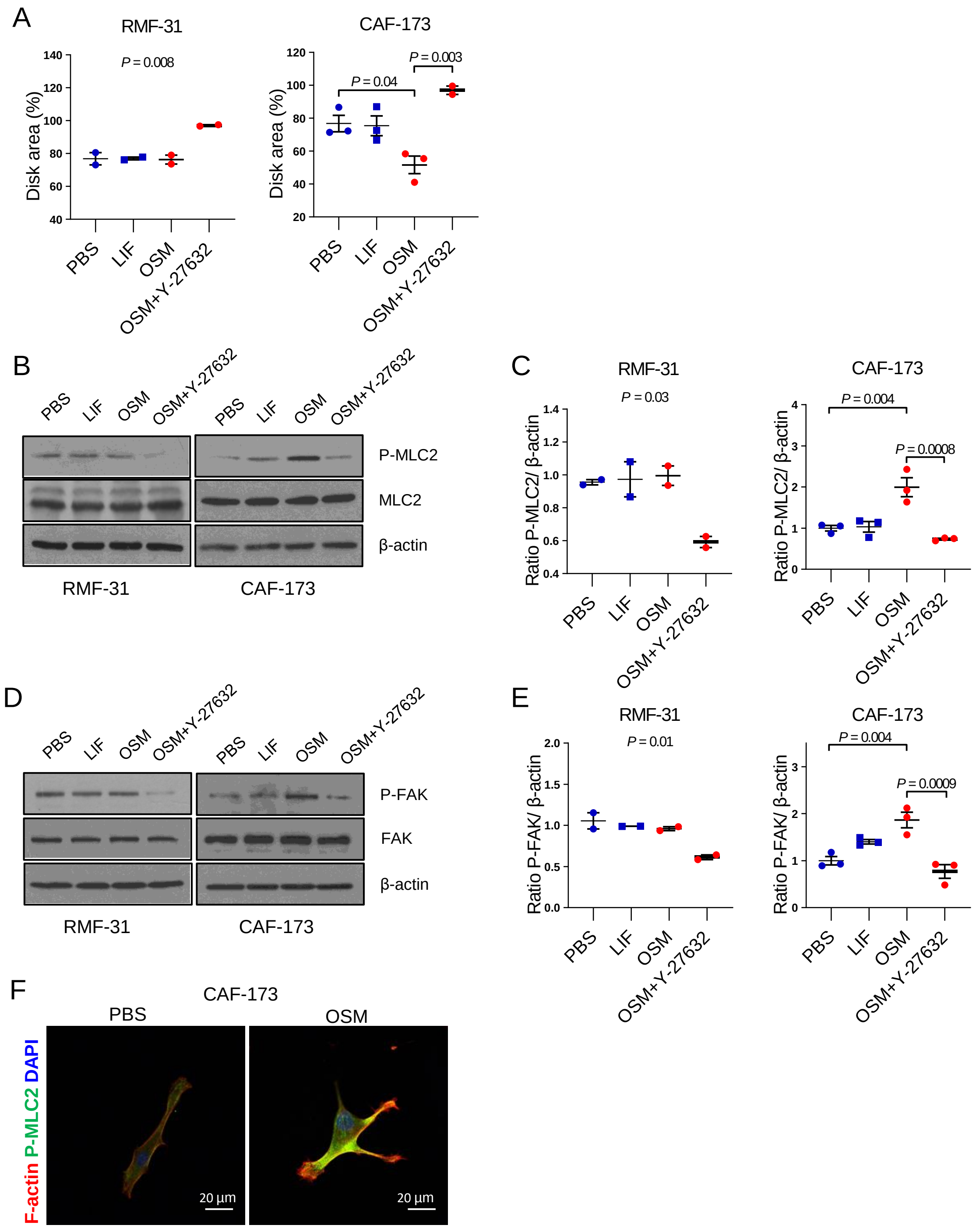

Supplemental Figure 6. Effects of OSM and LIF in the contractility and the cytoskeleton of Cancer Associated Fibroblasts (CAFs). (A) Quantification of disk areas from collagen contraction assays of RMF-31 and CAF-173 fibroblasts pre-treated with OSM, LIF or OSM + Y-27632. **(B-E)** Western blots (**B** and **D**) and densitometric analyses (**C** and **E**) of P-MLC2 (**B** and **C**) and P-FAK (**D** and **E**) protein levels in RMF-31 and CAF-173 treated with OSM, LIF or OSM + Y-27632. Total MLC2 and FAK blots were obtained in gels run in parallel. In **A**-**E**, 2 (for RMF-31) or 3 (for CAF-173) independent experiments were performed, and *P* values were calculated using one-way ANOVA with post Tukey's multiple comparison test. (**F**) Representative picture of F-actin and P-MLC2 immunofluorescence staining of CAF-173 treated with OSM. Scale bar is 20 mm.

Supplemental Figure 7

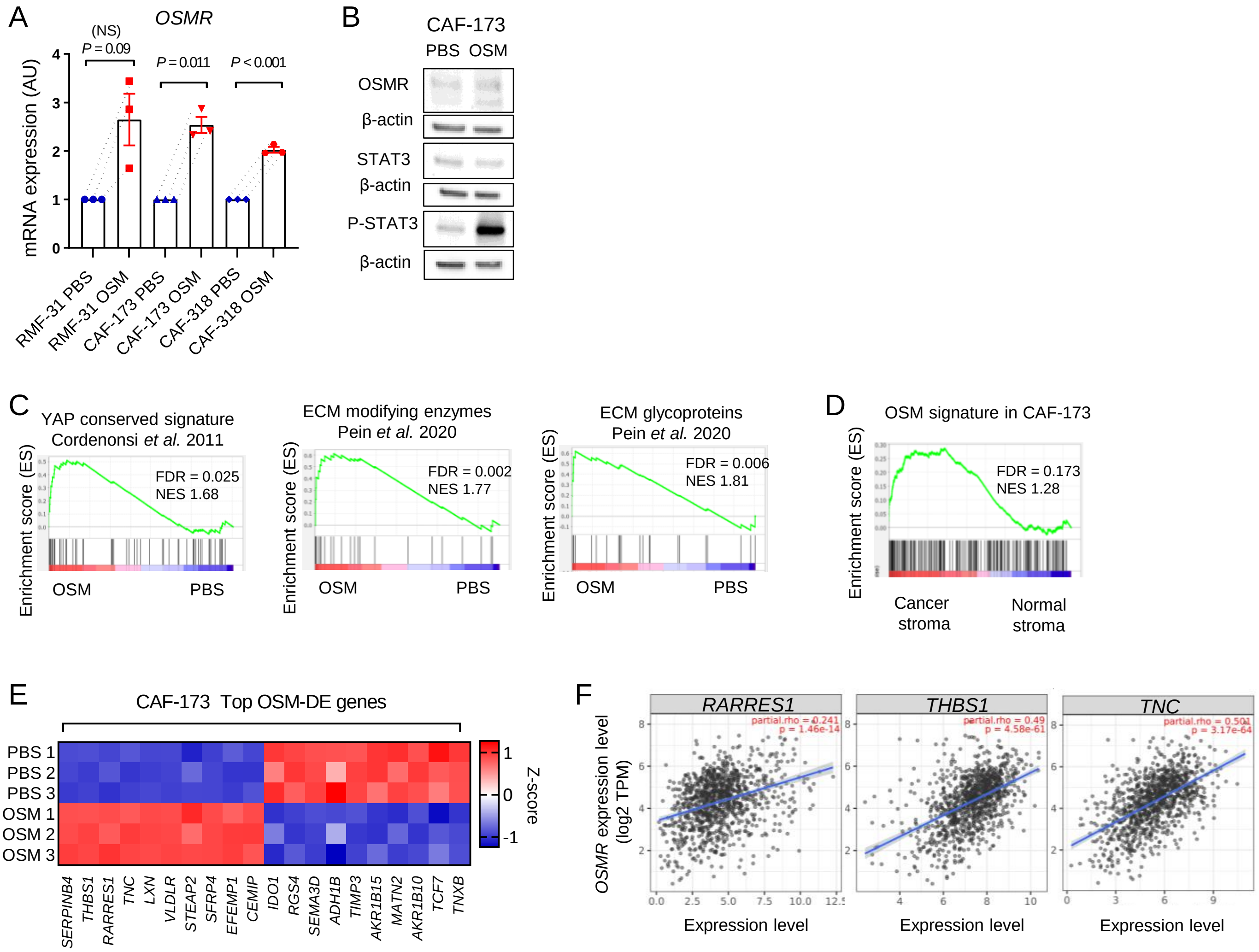

Supplemental Figure 7. Effects of OSM in the gene expression profile of Cancer Associated Fibroblasts (CAFs). (**A**) *OSMR* mRNA expression levels analyzed by RT-qPCR in 3D fibroblast spheres treated with OSM for 4 days. n=3 independent experiments. *P* values were calculated using paired two-tailed t test. (**B**) Western blot of OSMR, STAT3 and P-STAT3 protein levels in CAF-173 treated with OSM. One representative experiment of 4 performed is shown. (**C**) Gene set enrichment analysis (GSEA) showing enrichment of the indicated signatures in microarray data of CAF-173 CAFs treated with OSM. (**D**) GSEA showing enrichment of the signature composed of the 233 upregulated genes in CAF-173 cells treated with OSM, in microarray data of cancer and normal breast stroma from Finak et al. (2008) (GSE9014). (**E**) Top 10 up- and down-regulated genes in microarray data of CAF-173 treated with OSM for 4 days. DE: differentially expressed. (**F**) Correlation of *OSMR* mRNA levels with *RARRES1*, *THBS1* and *TNC* expression in breast cancer clinical samples, n=1100. Data were downloaded from TIMER web platform and Spearman correlation coefficients and *P* values are shown.

Supplemental Figure 8

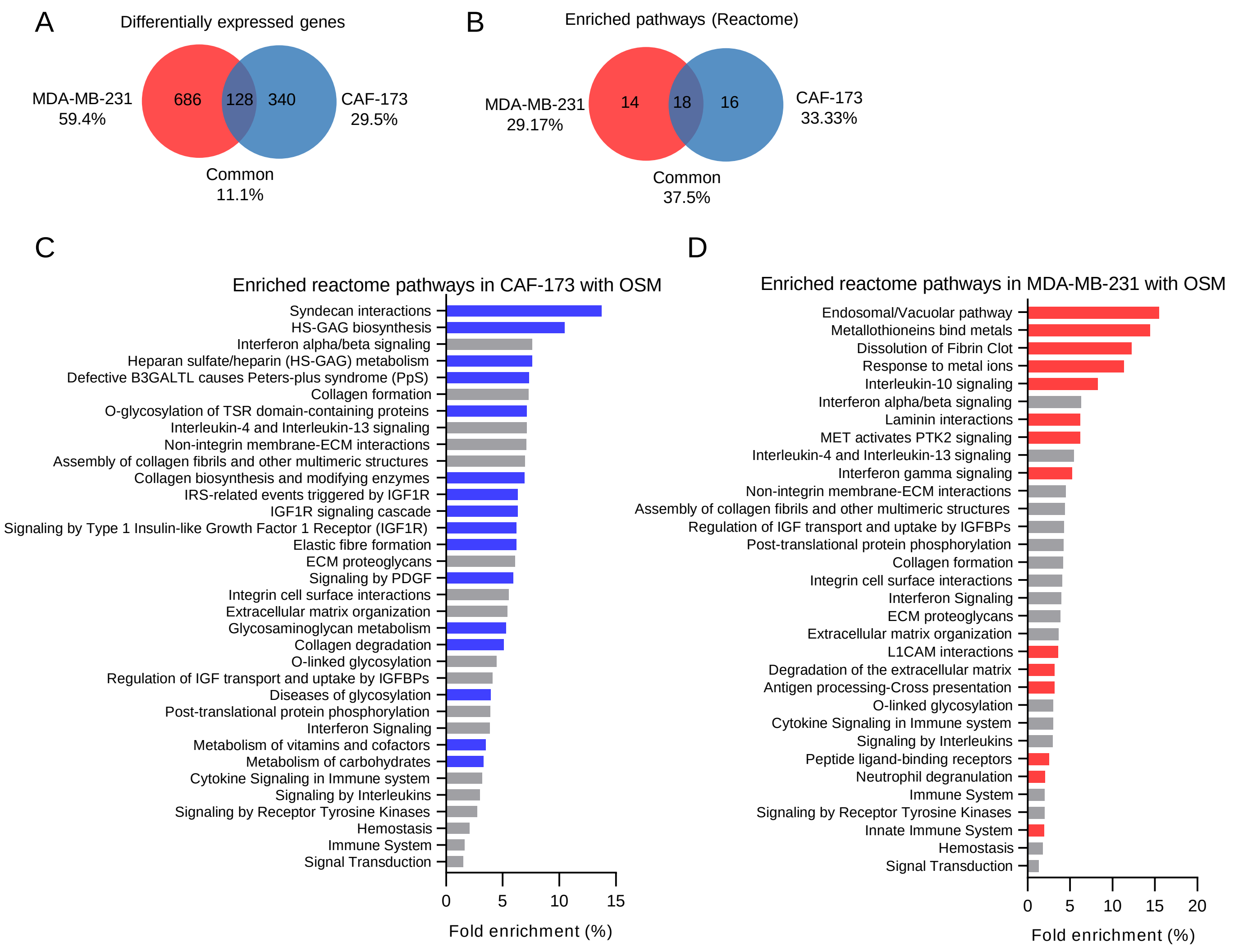

Supplemental Figure 8. Comparison of OSM-induced transcriptomic changes in MDA-MB-231 and CAF-173. (A and **B)** Venn diagrams showing differentially expressed genes (**A**) and enriched pathways (**B**) in CAF-173, MDA-MB-231 or both cell types activated by OSM (n=3 independent experiments). (**C** and **D**) Enriched pathways in transcriptomic data of CAF-173 (**C**) and MDA-MB-231 (**D**) cells activated by OSM. Blue, red and grey bars indicate pathways enriched by OSM only in CAF-173, only in MDA-MB-231 or in both cell types respectively.

Supplemental Figure 9

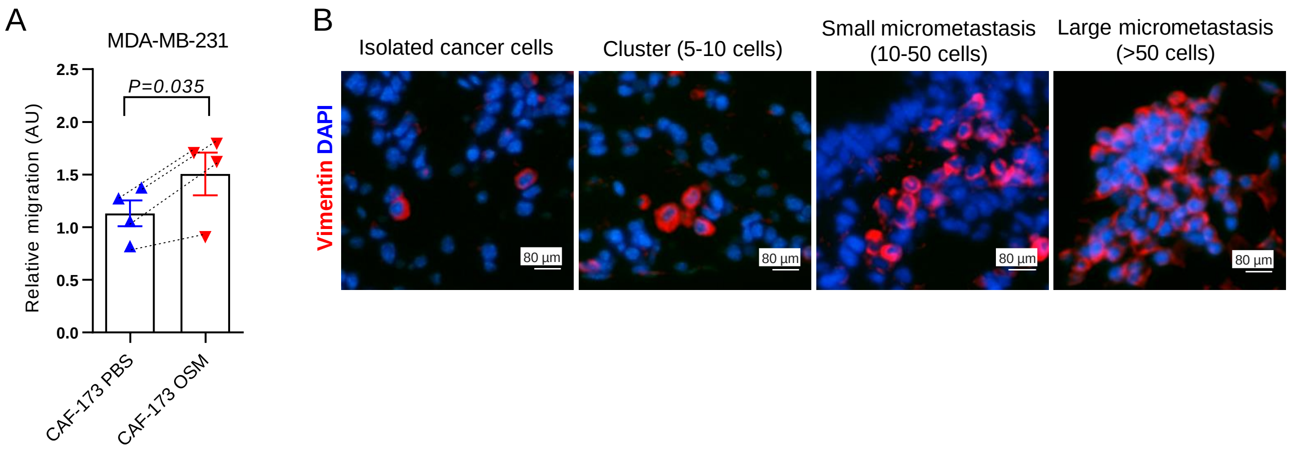

Supplemental Figure 9. Effect of OSM-activated CAF-173 on cancer cell migration and metastasis. (**A**) Effect of conditioned media from CAF-173 treated with PBS (Control) or OSM 10ng/mL for 72 hours on MDA-MB-231 migration, n=4 independent experiments. *P* value was determined using paired two tailed t test. (**B**) Representative pictures of vimentin immunofluorescence staining in lungs of mice (n=5) injected orthotopically with CAF-173 OSM + MDA-MB-231 described in Figure 6A and E. Scale bar is 80 mm.

Supplemental Figure 10

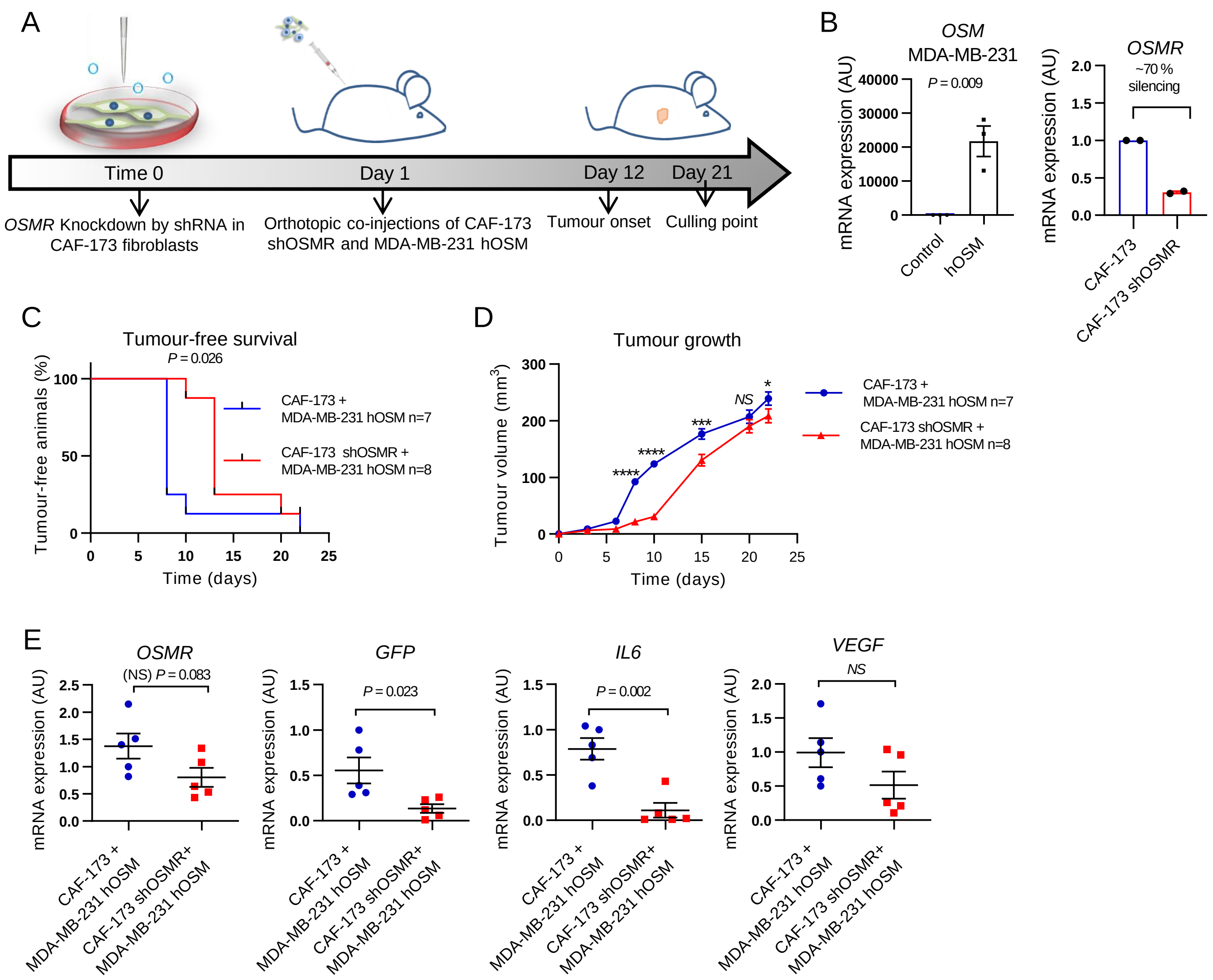

Supplemental Figure 10. Effect of OSMR knockdown in CAF-173 on tumour progression. (**A**) Experimental set-up of the in vivo experiment designed to assess the contribution to breast cancer progression of OSMR knockdown in fibroblasts. Control and shOSMR CAF-173 were co-injected with MDA-MB-231-hOSM (500,000 cells each cell line) in matrigel (1:1 ratio) in the mammary gland fat pad of nude mice. n=7 for control CAF-173 + MDA-MB-231 hOSM and n=8 for CAF-173 shOSMR +MDA-MB-231 hOSM. (**B**) *OSM* and *OSMR* mRNA expression levels analyzed by RT-qPCR in MDA-MB-231 and CAF-173 after hOSM and shOSMR plasmid transfection, respectively. (n=3 for *OSM* and n=2 for *OSMR*). (**C** and **D**) Kaplan-Meier curves for tumour-free survival (**C**) and tumour growth (**D**) of orthotopic tumours described in (**A**). (**E**) *OSMR*, *GFP*, *IL6* and *VEGF* mRNA expression levels analyzed by RT-qPCR in 5 tumours per experimental group, as described in (**A**). *P* value was calculated using paired (**B**) or unpaired (**E**) two tailed t test, the Mantel-Cox test (**C**) or two-way ANOVA with post Sidak's multiple comparison test (**D**). * *P* < 0,05; *** *P* < 0.001; **** *P* < 0.0001.

Supplemental Figure 11

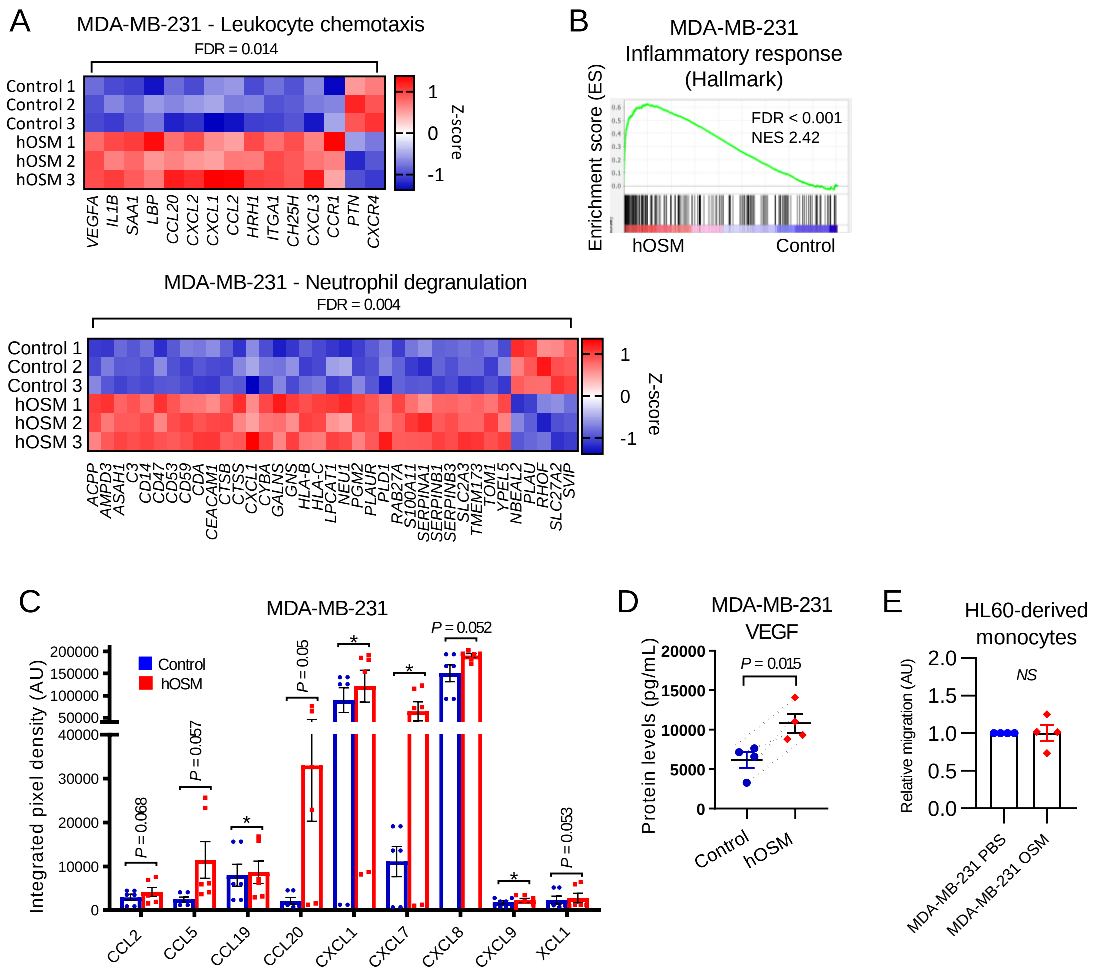

Supplemental Figure 11. OSM induces expression of chemokines in cancer cells. (**A**) Heatmap showing normalized mRNA expression of genes induced by OSM in MDA-MB-231 cancer cells and included in the indicated gene ontology (GO) pathways**.** (**B**) Gene set enrichment analysis (GSEA) showing enrichment of inflammatory hallmark signature in microarray expression data of MDA-MB-231 hOSM. ES: enrichment score; NES: normalized enrichment score. (**C** and **D**) Chemokine array analysis (**C**) and VEGF levels (**D**) in conditioned media from MDA-MB-231 h-OSM and control cells, 72h after seeding. n=6 (**C**) or n=4 (**D**) independent experiments. *P* values were determined using paired two-tailed t tests. * *P* < 0,05. (**E**) Effect of conditioned media from MDA-MB-231 treated with PBS (Control) or OSM 10ng/mL for 72 hours on HL-60 derived monocytes migration, n=4 independent experiments.

Supplemental Figure 12

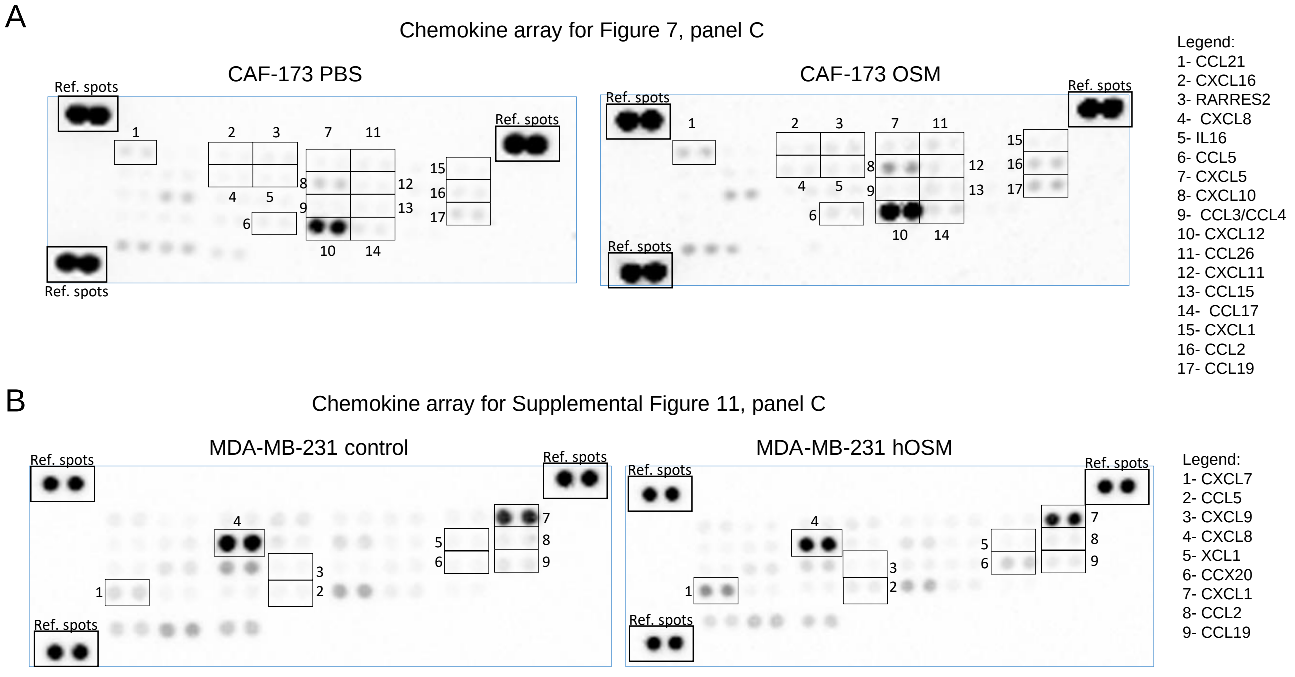

Supplemental Figure 12. Chemokine array experiments. (**A** and **B**) Representative images of chemokine array membranes of conditioned media experiments from CAF-173 (**A**) and MDA-MB-231 (**B**) shown in Figure 7C and Supplemental Figure 11C respectively. Ref. spots: Reference spots.

Supplemental Figure 13

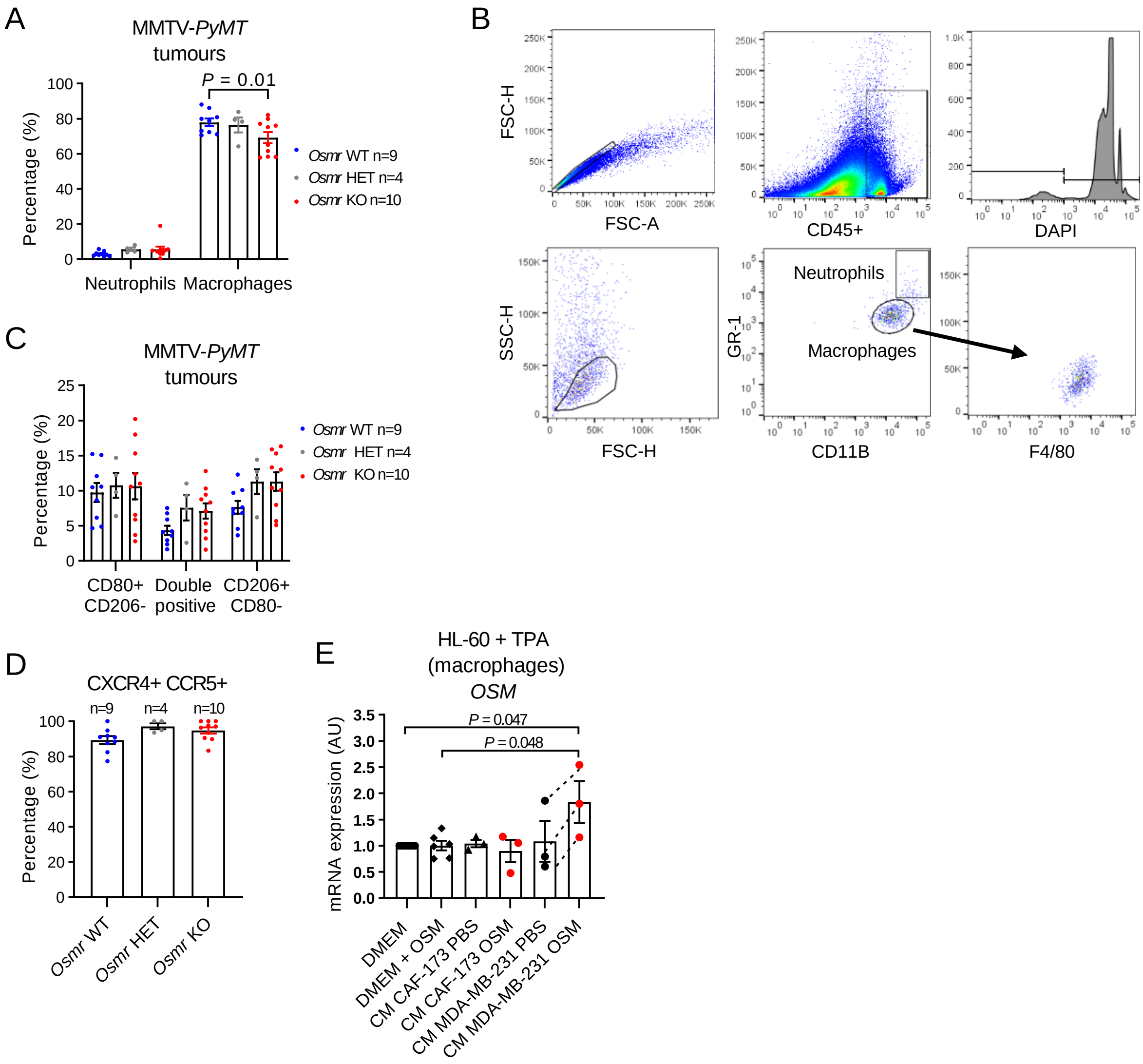

Supplemental Figure 13. Effect of OSM signalling on tumour infiltrating myeloid cells. (**A**-**D**) Percentages (**A**), gating strategy (**B**) and phenotypic characterization of macrophages (**C**) and neutrophils (**D**) infiltrating tumours from MMTV-*PyMT*:*Osmr* wild-type (WT), heterozygous (HET), and KO mice at 14 weeks of age by flow cytometry. (**E**) *OSM* mRNA levels in HL-60 + TPA (macrophages) treated with conditioned media (CM) from MDA-MB-231 and CAF-173 pre-treated with OSM 10 ng/mL for 72h. n=3 independent experiments, except for DMEM +OSM condition (n=6). *P* values were calculated using two-way ANOVA with post Dunnett's multiple comparison test (**A**) or one-way ANOVA with post Tukey's multiple comparison test (**E**).

Supplemental Figure 14

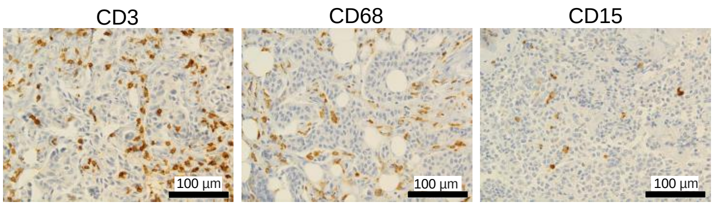

Supplemental Figure 14. Characterization of infiltrating immune cells in human breast cancer samples. Representative pictures of CD3, CD68 and CD15 staining in samples from breast cancer patients included in Figure 9. n=50, 38 and 52 cases stained for CD3, CD68 and CD15 respectively.

Supplemental Figure Legends

Supplemental Figure 1. Effects of OSM:OSMR signalling in cancer progression in the MMTV-*PyMT* preclinical model of breast cancer. (**A** and **B**) Western blot (**A**) and densitometric analysis (**B**) of OSMR protein levels in tumours at culling point from the different experimental groups of Figure 1A: MMTV-*PyMT:Osmr* wild-type (WT), MMTV-*PyMT:Osmr* heterozygous (HET), and MMTV-*PyMT:Osmr* knockout (KO) mice. (**C**-**E**) Tumour onset (**C**), representative pictures of whole mount staining of mammary glands at week 9 (**D**) and histopathological analysis of tumours at culling point (**E**) in the different experimental groups of Figure 1A. n= 12 WT, 6 HET, 5 KO in (**D**) and 21 WT, 20 HET, 15 KO in (**E**). (**F** and **G**) Representative pictures (**F**) and quantification (**G**) of Ki67 and active cleaved caspase 3 (cCaspase3) IHC staining in tumours at culling point of the different experimental groups of Figure 1A. Quantification was performed by manual counting of the percentage of positive tumour cells in a total of 8 pictures per tumour and 5 tumours per group. Scale bar is 20 μm (**E**) and 50 μm (**F**). *P* values were calculated using one-way ANOVA test (**B**, **C** and **G**).

Supplemental Figure 2. Effects of stromal OSMR deletion in syngeneic models of breast cancer. (**A**) Western blot of OSMR, STAT3 and P-STAT3 protein levels in TS1 cells treated with 10 and 100 ng/mL of recombinant murine OSM for 24h. (**B** and **C**) Western blot (**B**) and densitometric analysis (**C**) of OSMR protein levels in TS1 derived-tumours from animals *Osmr* wild-type (WT, n=8) or knockout (KO, n=6) injected orthotopically with TS1 cells (experiment 2, Figure 2, A-E). OSMR expression in tumours from *Osmr* KO animals derives from the TS1 cancer cell compartment. (**D**) Experimental set-up of the in vivo experiment designed to assess the importance of OSMR signalling in cancer cells and in the tumour microenvironment, in which control and *Osmr* KO TS1 cells were orthotopically injected into the mammary fat pad of *Osmr* WT and KO mice. n=3 Osmr WT mice injected with control cells, n=7 Osmr WT mice injected with Osmr KO cells, n=5 Osmr KO mice injected with control cells, and n=7 Osmr KO mice

injected with Osmr KO cells. (**E**) Western Blot of OSMR protein levels in TS1 parental, control and *Osmr* KO cells. (**F** and **G**) Kaplan-Meier curves for tumour-free survival (**F**) and final tumour volume (**G**) after dissection of orthotopic tumours described in (**D**). In **A** and **E**, one representative experiment of 2 performed is shown. *P* values were calculated using unpaired two-tailed t test (**C**), the Mantel-Cox test (**F**) or one-way ANOVA test (**G**).

Supplemental Figure 3. Expression of OSM and OSMR in clinical samples from multiple cancer types. (**A**) *OSM* and *OSMR* mRNA expression in normal stroma versus cancer stroma samples of colorectal and ovarian cancer. Data were downloaded from GEO DataSets (GSE35602 and GSE40595). *P* values were calculated using unpaired two-tailed t test. (**B**) Kaplan-Meier curves showing disease-free survival (DFS) for breast cancer patients with high and low *OSM* expression, included in the METABRIC and Wang datasets. (**C**) Kaplan-Meier curves showing overall survival for cancer patients of the indicated tumour type with high and low *OSM* expression. Data were obtained using KM plotter website. In **B** and **C**, *P* values were determined using the Mantel-Cox test, and high and low *OSM* levels were stratified by median values.

Supplemental Figure 4. *Osm* and *Osmr* expression in murine tumours. (**A** and **B**) Experimental set-up (**A**) and gating strategy (**B**) for FACS sorting experiments of TS1 orthotopic tumours. (**C** and **D**) *Osm* and *Osmr* mRNA expression levels analyzed by RT-qPCR of FACS sorted populations of TS1 orthotopic tumours (**C**) or MMTV-*PyMT* FACS sorted tumours described in Ferrari et al. (2019) (**D**). In **D**, *P* values were determined using one-way ANOVA test. In **A** and **C**, the two different experiments were performed independently, each one in a pool of 4 tumours from individual animals. Graphs represent mean of 2 technical replicates (**C**) or of 6 different tumours (**D**).

Supplemental Figure 5. *OSM* and *OSMR* expression in human cell lines and tumours. (**A**) mRNA expression levels of the indicated IL6 family members and associated receptors

analyzed by RT-qPCR in MDA-MB-231 breast cancer cells, and undifferentiated and TPA-differentiated HL-60 cells. n=3 independent experiments. (**B**) *OSM* and *OSMR* mRNA relative values in a panel of 69 human cell lines from multiple anatomical sites. Data were downloaded from Human Protein Atlas. (**C**) *OSM* and *OSMR* mRNA expression in epithelial cell (Epcam$^+$), immune cell (CD45$^+$), fibroblast (FAP$^+$) and endothelial cell (CD31$^+$) FACS-sorted populations from colorectal cancer samples (n=6). Boxplot with Tukey whiskers is shown, bounds of the boxes represent the 25th and 75th percentiles, and lines within the boxes indicate the median. Data were downloaded from GSE39396 GEO DataSets. *P* values were determined using one-way ANOVA test.

Supplemental Figure 6. Effects of OSM and LIF in the contractility and the cytoskeleton of Cancer Associated Fibroblasts (CAFs). (A) Quantification of disk areas from collagen contraction assays of RMF-31 and CAF-173 fibroblasts pre-treated with OSM, LIF or OSM + Y-27632. **(B**-**E)** Western blots (**B** and **D**) and densitometric analyses (**C** and **E**) of P-MLC2 (**B** and **C**) and P-FAK (**D** and **E**) protein levels in RMF-31 and CAF-173 treated with OSM, LIF or OSM + Y-27632. Total MLC2 and FAK blots were obtained in gels run in parallel. In **A**-**E**, 2 (for RMF-31) or 3 (for CAF-173) independent experiments were performed, and *P* values were calculated using one-way ANOVA with post Tukey's multiple comparison test. (**F**) Representative picture of F-actin and P-MLC2 immunofluorescence staining of CAF-173 treated with OSM. Scale bar is 20 μm.

Supplemental Figure 7. Effects of OSM in the gene expression profile of Cancer Associated Fibroblasts (CAFs). (**A**) *OSMR* mRNA expression levels analyzed by RT-qPCR in 3D fibroblast spheres treated with OSM for 4 days. n=3 independent experiments. *P* values were calculated using paired two-tailed t test. (**B**) Western blot of OSMR, STAT3 and P-STAT3 protein levels in CAF-173 treated with OSM. One representative experiment of 4 performed is shown. (**C**) Gene set enrichment analysis (GSEA) showing enrichment of the indicated signatures in microarray

data of CAF-173 CAFs treated with OSM. (**D**) GSEA showing enrichment of the signature composed of the 233 upregulated genes in CAF-173 cells treated with OSM, in microarray data of cancer and normal breast stroma from Finak et al. (2008) (GSE9014). (**E**) Top 10 up- and down-regulated genes in microarray data of CAF-173 treated with OSM for 4 days. DE: differentially expressed. (**F**) Correlation of *OSMR* mRNA levels with *RARRES1*, *THBS1* and *TNC* expression in breast cancer clinical samples, n=1100. Data were downloaded from TIMER web platform and Spearman correlation coefficients and *P* values are shown.

Supplemental Figure 8. Comparison of OSM-induced transcriptomic changes in MDA-MB-231 and CAF-173. (A and **B)** Venn diagrams showing differentially expressed genes (**A**) and enriched pathways (**B**) in CAF-173, MDA-MB-231 or both cell types activated by OSM (n=3 independent experiments). (**C** and **D**) Enriched pathways in transcriptomic data of CAF-173 (**C**) and MDA-MB-231 (**D**) cells activated by OSM. Blue, red and grey bars indicate pathways enriched by OSM only in CAF-173, only in MDA-MB-231 or in both cell types respectively.

Supplemental Figure 9. Effect of OSM-activated CAF-173 on cancer cell migration and metastasis. (**A**) Effect of conditioned media from CAF-173 treated with PBS (Control) or OSM 10ng/mL for 72 hours on MDA-MB-231 migration, n=4 independent experiments. *P* value was determined using paired two tailed t test. (**B**) Representative pictures of vimentin immunofluorescence staining in lungs of mice (n=5) injected orthotopically with CAF-173 OSM + MDA-MB-231 described in Figure 6A and E. Scale bar is 80 μm.

Supplemental Figure 10. Effect of OSMR knockdown in CAF-173 on tumour progression. (**A**) Experimental set-up of the in vivo experiment designed to assess the contribution to breast cancer progression of OSMR knockdown in fibroblasts. Control and shOSMR CAF-173 were co-injected with MDA-MB-231-hOSM (500,000 cells each cell line) in matrigel (1:1 ratio) in the mammary gland fat pad of nude mice. n=7 for control CAF-173 + MDA-MB-231 hOSM and n=8 for CAF-173 shOSMR +MDA-MB-231 hOSM. (**B**) *OSM* and *OSMR* mRNA expression levels

analyzed by RT-qPCR in MDA-MB-231 and CAF-173 after hOSM and shOSMR plasmid transfection, respectively. (n=3 for *OSM* and n=2 for *OSMR*). (**C** and **D**) Kaplan-Meier curves for tumour-free survival (**C**) and tumour growth (**D**) of orthotopic tumours described in (**A**). (**E**) *OSMR*, *GFP*, *IL6* and *VEGF* mRNA expression levels analyzed by RT-qPCR in 5 tumours per experimental group, as described in (**A**). *P* value was calculated using paired (**B**) or unpaired (**E**) two tailed t test, the Mantel-Cox test (**C**) or two-way ANOVA with post Sidak's multiple comparison test (**D**). * $P < 0,05$; *** $P < 0.001$; **** $P < 0.0001$.

Supplemental Figure 11. OSM induces expression of chemokines in cancer cells. (**A**) Heatmap showing normalized mRNA expression of genes induced by OSM in MDA-MB-231 cancer cells and included in the indicated gene ontology (GO) pathways**.** (**B**) Gene set enrichment analysis (GSEA) showing enrichment of inflammatory hallmark signature in microarray expression data of MDA-MB-231 hOSM. ES: enrichment score; NES: normalized enrichment score. (**C** and **D**) Chemokine array analysis (**C**) and VEGF levels (**D**) in conditioned media from MDA-MB-231 h-OSM and control cells, 72h after seeding. n=6 (**C**) or n=4 (**D**) independent experiments. *P* values were determined using paired two-tailed t tests. * $P < 0,05$. (**E**) Effect of conditioned media from MDA-MB-231 treated with PBS (Control) or OSM 10ng/mL for 72 hours on HL-60 derived monocytes migration, n=4 independent experiments.

Supplemental Figure 12. Chemokine array experiments. (**A** and **B**) Representative images of chemokine array membranes of conditioned media experiments from CAF-173 (**A**) and MDA-MB-231 (**B**) shown in Figure 7C and Supplemental Figure 11C respectively. Ref. spots: Reference spots.

Supplemental Figure 13. Effect of OSM signalling on tumour infiltrating myeloid cells. (**A**-**D**) Percentages (**A**), gating strategy (**B**) and phenotypic characterization of macrophages (**C**) and neutrophils (**D**) infiltrating tumours from MMTV-*PyMT*:*Osmr* wild-type (WT), heterozygous (HET), and KO mice at 14 weeks of age by flow cytometry. (**E**) *OSM* mRNA levels in HL-60 + TPA

(macrophages) treated with conditioned media (CM) from MDA-MB-231 and CAF-173 pre-treated with OSM 10 ng/mL for 72h. n=3 independent experiments, except for DMEM +OSM condition (n=6). *P* values were calculated using two-way ANOVA with post Dunnett's multiple comparison test (**A**) or one-way ANOVA with post Tukey's multiple comparison test (**E**).

Supplemental Figure 14. Characterization of infiltrating immune cells in human breast cancer samples. Representative pictures of CD3, CD68 and CD15 staining in samples from breast cancer patients included in Figure 9. n=50, 38 and 52 cases stained for CD3, CD68 and CD15 respectively.

Supplemental Table 2: Antibodies used in this study. IF: immunofluorescence, RTU: ready to use

Application	Antibody	Company	Reference	Dilution
Western blot	beta-actin	Sigma	A5441	1/2000
Western blot	FN	Abcam	AB2413	1/1000
Western blot	OSMR (mouse)	R&D Systems	AF662	1/500
Western blot	OSMR (human)	Santa Cruz	30010	1/500
Western blot	P-STAT3 (Y705)	Cell Signaling	9145	1/2000
Western blot	STAT3	Cell Signaling	9139	1/1000
Western blot and IF	P-MLC2 (S19)	Cell Signaling	3671	1/1000 – 1/200
Western blot	MLC2	Cell Signaling	3672	1/1000
Western blot	P-FAK (Y397)	Cell Signaling	8556	1/1000
Western blot	FAK	Cell Signaling	3285	1/1000
Western blot	Rabbit IgG HRP linked whole Ab	GE Healthcare (Thermo Fisher)	NA934	1/2000
Western blot	Mouse IgG HRP linked whole Ab	GE Healthcare (Thermo Fisher)	NA931	1/2000
Western blot	Rabbit Anti-Goat IgG H&L (HRP)	Abcam	ab97100	1/2000
FACS	CD11b-FITC	BD Biosciences	561688	1/50
FACS	CD3-PE	Thermo Fisher	12-0031-82	1/40
FACS	CD31-FITC	MACS	130-097-418	1/10
FACS	CD45-PerCP/Cy5.5	BioLegend	10313	1/80
FACS	EPCAM-APC	Biolegend	118214	1/80
FACS	CD45-BV480	BD Biosciences	566077	1/200
FACS	GR1-BV711	Biolegend	108443	1/200
FACS	CD11B-BUV805	BD Biosciences	612977	1/200
FACS	F4/80-BUV661	BD Biosciences	750643	1/200
FACS	CD206-AlexaFluor647	BD Biosciences	565250	1/200
FACS	CD80-PE	BD Biosciences	553769	1/200
FACS	CXCR4-PE	Biolegend	146505	1/200
FACS	CCR5-APC	Biolegend	107011	1/200
Immunofluorescence	Vimentin	Cell Signaling	5741	1/100
Immunofluorescence	Goat anti-rabbit IgG(H+L) Alexa fluor Plus 555	Invitrogen	A32732	1/500
Immunofluorescence	Goat anti-rabbit IgG(H+L) Alexa fluor 488	Invitrogen	A11008	1/500
Immunohistochemistry	Cleaved Caspase 3	R&D Systems	AF835	1/1000
Immunohistochemistry	F4/80	Bio-Rad	MCA497GA	1/10
Immunohistochemistry	Ki67	Novocastra	ACK02	1/800
Immunohistochemistry	Ly6G	BD Biosciences	551459	1/70

Immunohistochemistry	Goat Anti-Rabbit IgG biotinyated	Vector Laboratories	BA-1000	1/400
Immunohistochemistry	Goat Anti-Rat IgG biotinyated	Vector Laboratories	BA-94001	1/400
Immunohistochemistry	Goat Anti-Mouse IgG biotinyated	Vector Laboratories	BA-920	1/400
Immunohistochemistry	OSM	Sigma-Aldrich	HPA029814	1/50
Immunohistochemistry	CD3	Ventana	790-4341	RTU
Immunohistochemistry	CD15	Ventana	760-2504	RTU
Immunohistochemistry	CD68	Dako	IR613	RTU

Supplemental Table 3: qPCR primers used in this study.

Gene	Species	Forward primer	Reverse primer
Alu	Human	ACGCCTGTAATCCCAGCACTT	TCGCCCAGGCTGGAGTGCA
FAP	Human	CAAAGGCTGGAGCTAAGAATCC	ACTGCAAACATACTCGTTCATCA
IL6ST	Human	AGGACCAAAGATGCCTCAAC	GAATGAAGATCGGGTGGATG
HMBS	Human	GGCAATGCGGCTGCAA	GGGTACCCACGCGAATCAC
IL6	Human	CCAGGAGCCCAGCTATGAAC	CCCAGGGAGAAGGCAACTG
IL6R	Human	CCCCTCAGCAATGTTGTTTGT	CTCCGGGACTGCTAACTGG
LIF	Human	CCAACGTGACGGACTTCCC	TACACGACTATGCGGTACAGC
LIFR	Human	TGGAACGACAGGGGTTCAGT	GAGTTGTGTTGTGGGTCACTAA
OSM	Human	CTCGAAAGAGTACCGCGTG	TCAGTTTAGGAACATCCAGGC
OSMR	Human	AATGTCAGTGAAGGCATGAAAGG	GAAGGTTGTTTAGACCACCCC
POSTN	Human	CTCATAGTCGTATCAGGGGTCG	ACACAGTCGTTTTCTGTCCAC
VEGF	Human	AGGGCAGAATCATCACGAAGT	AGGGTCTCGATTGGATGGCA
18S	Human and mouse	CGCGGTTCTATTTTGTTGGT	CGGTCCAAGAATTTCACCTC
HPRT	Human and mouse	TGACACTGGCAAAACAATGCA	GGTCCTTTTCACCAGCAAGCT
Actb	Mouse	GCTACAGCTTCACCACCACA	TCTCCAGGGAGGAAGAGGAT
Osm	Mouse	ATGCAGACACGGCTTCTAAGA	TTGGAGCAGCCACGATTGG
Osmr	Mouse	CATCCCGAAGCGAAGTCTTGG	GGCTGGGACAGTCCATTCTAAA
GFP		CTAGGCCACAGAATTGAAAGATCT	GTAGGTGGAAATTCTAGCATCATC C

Supplemental Table 4: Gene list for the fibroblast activation markers signature, manually curated from Sahai et al. 2020, used in Figure 5F.

TNC	*COL5A1*	*IL6*	*VEGFA*	*CCN2*
HGF	*CCN1*	*POSTN*	*ACTA2*	*CXCL12*
CXCL9	*PDGFRA*	*FAP*	*IL1B*	*FGF5*
GAS6	*COL1A2*	*LIF*	*PTK2*	*GDF15*
PDGFA	*HSF1*	*INHBA*	*TGFB1*	*GRN*
VIM	*ILIA*	*S100A4*		

Supplemental Table 5: Gene list for the OSM-induced signature in CAF-173 used in Supplemental Figure 7D.

SERPINB4	*PPAP2B*	*CRISPLD2*	*SUSD6*	*POSTN*
THBS1	*STEAP1B*	*CXCL12*	*PFKFB4*	*EFCAB13*
LXN	*CHI3L1*	*SERPINB2*	*MOSPD1*	*B4GALT5*
RARRES1	*OSMR*	*FGF2*	*C10orf10*	*ABHD17C*
TNC	*SLC2A5*	*RHOBTB3*	*CTSL*	*MME*
VLDLR	*GPC6*	*ENG*	*NDST2*	*SERPINH1*
SFRP4	*TGFBI*	*FLNB*	*NPTX2*	*TSHZ3*
CEMIP	*SULF1*	*MARCH3*	*PRG4*	*RGS16*
ITGB3	*STEAP1*	*GALNT12*	*BICC1*	*ZDHHC9*
MYB	*JAK2*	*GPC6*	*WARS*	*HOXA4*
F2RL1	*IL13RA1*	*PUM3*	*PCED1A*	*LAMA4*
EFEMP1	*SLC2A14*	*LTBP2*	*NAMPT*	*NPC1*
KIF26B	*ACVR1B*	*P4HA1*	*DUSP1*	*VWA5A*
BRINP1	*CCDC71L*	*CPD*	*RUNX2*	*POLR2H*
SERPINE1	*SPRY1*	*NAV1*	*POLM*	*SMG6*
STEAP2	*MASP1*	*TRIB2*	*PREB*	*HEG1*
GJA1	*PDK1*	*ADAM19*	*PTGS2*	*GLT8D2*
DHRS3	*NREP*	*WISP1*	*SLC16A3*	*PTX3*
SEL1L3	*PDP1*	*ARRDC4*	*CYR61*	*C1R*
MYC	*SLC2A3*	*PTPN2*	*RNF144A*	*SYTL2*
IL1R1	*GYS1*	*ZPLD1*	*NXF3*	*SNAI1*
RDH10	*TMTC2*	*XYLT1*	*ADAMTS4*	*CDKN1A*
SERPINB7	*NRP2*	*KCTD10*	*ADAM12*	*TGFBR1*
COL5A1	*PCBP3*	*GPAM*	*CLCA2*	*ERO1A*
PLOD2	*BCL6*	*RP11-351M8.2*	*PGM3*	*DLC1*

FGF7	*ADM*	*C1QTNF6*	*LCE2A*	*JAK2*
SERPINB3	*IL6*	*CTGF*	*SLC22A23*	*DENND1B*
LRRC15	*MGP*	*SNX10*	*IGF1R*	*FHL2*
WWC1	*SDC1*	*BGN*	*BNIP3*	*ALDH1L2*
NID2	*FAM20C*	*FCHSD2*	*TMEM45A*	*CFAP54*
ITGB8	*MTHFD2*	*DAB2*	*JUNB*	*DNMT3B*
NNMT	*OSR1*	*TXNIP*	*FNIP2*	*SLC16A7*
HMOX1	*RAI14*	*GABRE*	*MICAL2*	*OPN3*
VEGFA	*RCAN2*	*RCL1*	*SMYD3*	*SRPX*
C2orf83	*AK4*	*FAM60A*	*HAS2*	*DMGDH*
SOCS3	*PFKFB3*	*IL4R*	*HS3ST3A1*	*BHLHE40*
PRDM1	*SBNO2*	*ELL2*	*TNS3*	*GK*
ARHGAP20	*NABP1*	*F3*	*CCL2*	*CAMK2N1*
DDIT4	*PLPP3*	*LOX*	*GALNT2*	*RASD1*
COLEC12	*GLIS3*	*COL5A2*	*SMIM3*	*NTNG1*
TMOD1	*TUBB3*	*AGTRAP*	*SOS1*	*ADAMTS3*
HS3ST3B1	*PLPP4*	*FAM20A*	*PAPPA*	*HLA-DQB1*
ARL4C	*PVR*	*AGFG1*	*GFPT2*	*KLF9*
IL33	*PGK1*	*PDGFRA*	*NRXN3*	*EPB42*
FNDC1	*THSD4*	*THBS2*	*CTBP2*	*STX19*
TAGLN	*HIF1A*	*COL8A1*	*C1orf158*	
TNFRSF10D	*NCAM2*	*PLAT*	*TMEM2*	

Supplemental Table 6: Histopathological information of breast cancer cases included in the TMAs. pT, pN and pM stand for pathological (p) tumour size (T), nodal status (N) and metastatic status (M) according to AJCC - TNM classification (8th Edition).

Clinicopathological feature	Category	Frequency N (%)
Age	Mean (range)	62 (27-92)
pT stage	pT1<br>pT2<br>pT3<br>pT4	58 (41 %)<br>69 (49 %)<br>5 (4 %)<br>9 (6 %)
pN stage	pN0<br>pN1<br>pN2<br>pN3<br>pNx	77 (55 %)<br>37 (26 %)<br>13 (9 %)<br>8 (6 %)<br>6 (4 %)
pM stage	0	141 (100 %)
Tumour grade	G1<br>G2<br>G3	15 (11 %)<br>61 (43 %)<br>65 (46 %)
Molecular subtype	Triple negative<br>HER2 positive<br>ER positive (luminal A,B)	27 (19 %)<br>32 (23 %)<br>82 (58 %)
Survival time (months)	Mean (range)	71 (1-233)
5-year survival rate (95%CI)	Survival rate in %	77% (71%-85%)

Full unedited gels for western blots

Full unedited gels for Figure 1, panel F

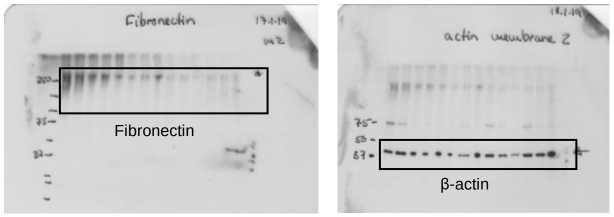

Full unedited gels for Supplemental Figure 1, panel A

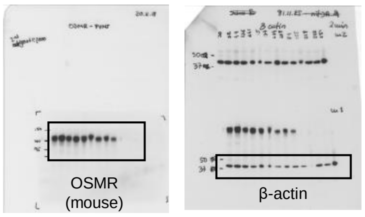

Full unedited gels for Supplemental Figure 2, panel A

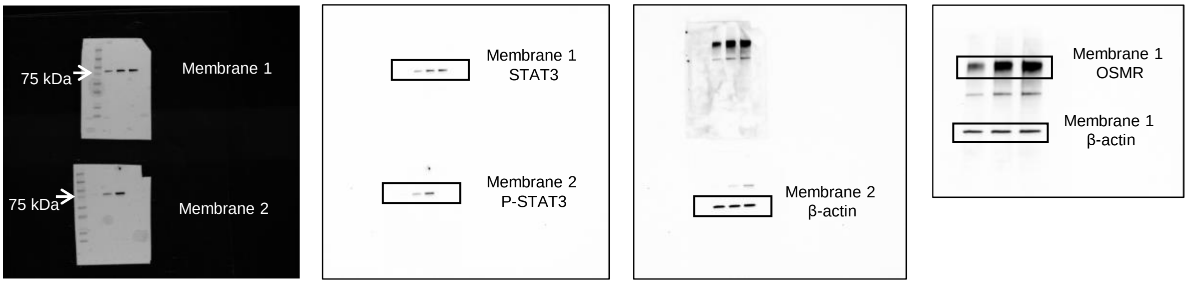

Full unedited gels for Supplemental Figure 2, panel B

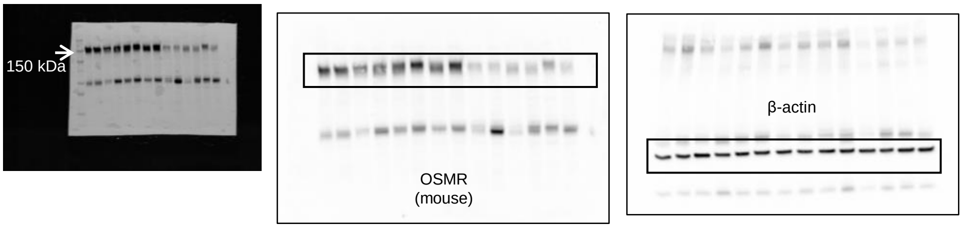

Full unedited gels for Supplemental Figure 2, panel E

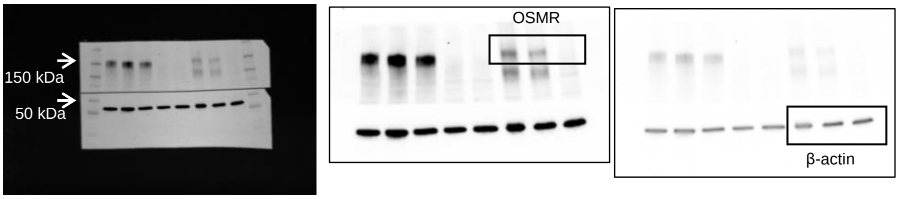

Full unedited gels for Supplemental Figure 6, panel B

RMF-31

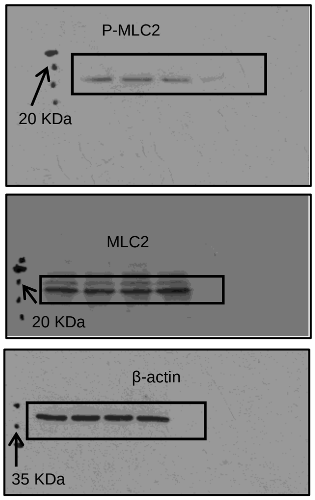

CAF-173

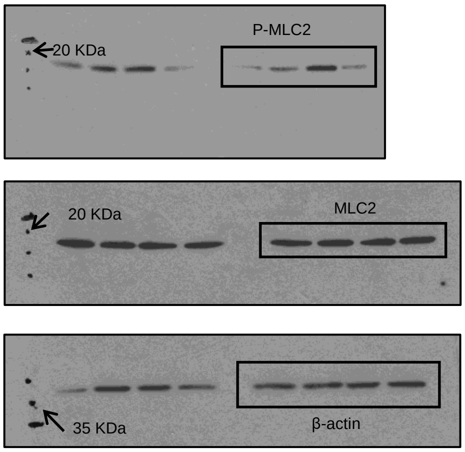

Full unedited gels for western blots

Full unedited gels for Supplemental Figure 6, panel D

RMF-31 CAF-173

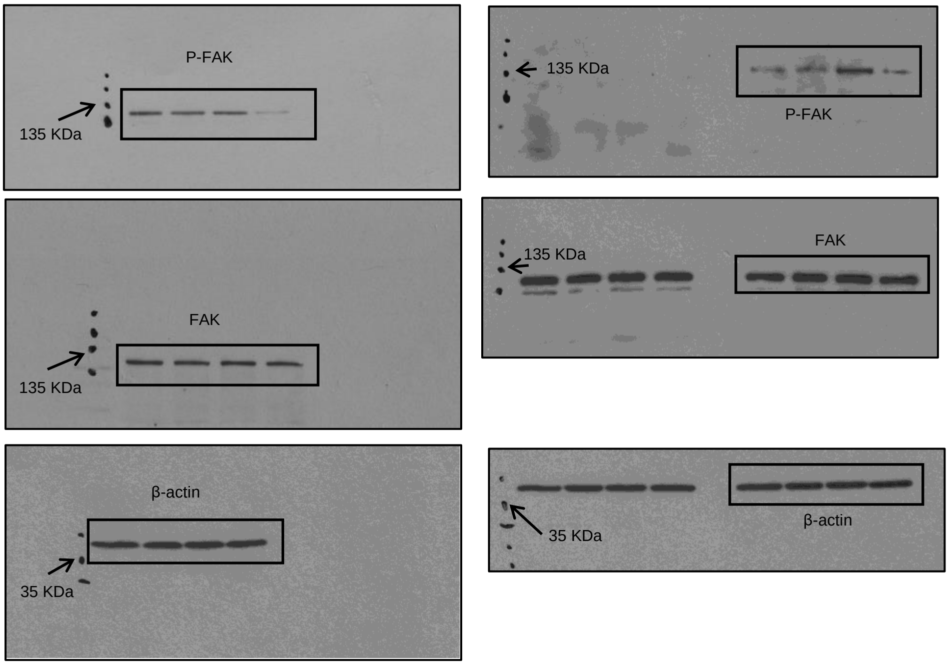

Full unedited gels for Supplemental Figure 7, panel B

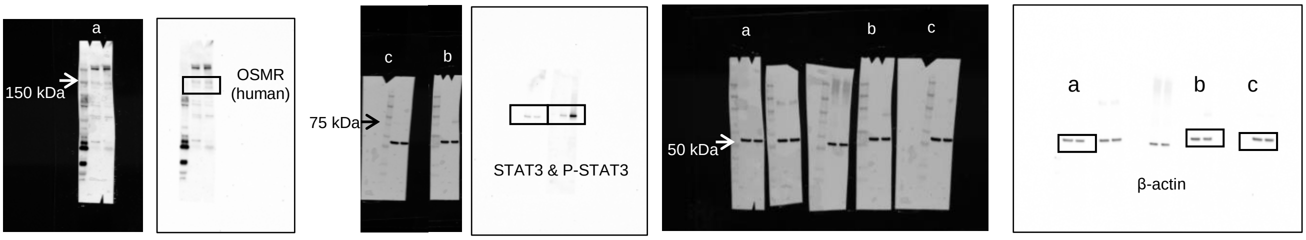

Supplement: Supplemental data [file jci-132-148667-s117.pdf]
